# Anti-integrin αvβ6 autoantibody in primary sclerosing cholangitis: a Japanese nationwide study

**DOI:** 10.1007/s00535-024-02169-w

**Published:** 2024-11-16

**Authors:** Muneji Yasuda, Masahiro Shiokawa, Takeshi Kuwada, Yoshihiro Nishikawa, Risa Nakanishi, Ikuhisa Takimoto, Koki Chikugo, Masataka Yokode, Yuya Muramoto, Shimpei Matsumoto, Takeharu Nakamura, Sakiko Ota, Tomoaki Matsumori, Keiko Kuroda, Takahisa Hachiya, Hajime Yamazaki, Norimitsu Uza, Yuzo Kodama, Tsutomu Chiba, Toshio Fujisawa, Atsumasa Komori, Masanori Abe, Izumi Yamaguchi, Fumihiko Matsuda, Hiroyuki Isayama, Atsushi Tanaka, Hiroshi Seno, on behalf of the Japan PSC Study Group (JPSCSG)

**Affiliations:** 1 Department of Gastroenterology and Hepatology, Kyoto University Graduate School of Medicine, Kyoto, Japan; 2 Medical and Biological, Laboratories Co., Ltd., Nagoya, Japan; 3 Section of Clinical Epidemiology, Department of Community Medicine, Graduate School of Medicine, Kyoto University, Kyoto, Japan; 4 Division of Gastroenterology, Department of Internal Medicine, Kobe University Graduate School of Medicine, Hyogo, Japan; 5 Kansai Electric Power Hospital, Osaka, Japan; 6 Department of Gastroenterology, Juntendo University Graduate School of Medicine, Tokyo, Japan; 7 Clinical Research Center, NHO Nagasaki Medical Center, Nagasaki, Japan; 8 Department of Gastroenterology and Metabology, Ehime University Graduate School of Medicine, Ehime, Japan; 9 Center for Genomic Medicine, Kyoto University Graduate School of Medicine, Kyoto, Japan; 10 Department of Medicine, Teikyo University School of Medicine, Tokyo, Japan

**Keywords:** Primary sclerosing cholangitis, Inflammatory bowel disease, Autoantibody

## Abstract

**Background:**

Although specific biomarkers for primary sclerosing cholangitis (PSC) are required, no such biomarkers have been identified. We previously reported that patients with PSC had anti-integrin αvβ6 autoantibodies at only two hospitals. In this study, we aimed to validate the accuracy of the autoantibodies in diagnosing PSC using the newly developed Anti-integrin αvβ6 enzyme-linked immunosorbent assay (ELISA) Kit, which enables quantitation and comparison of antibodies among different facilities.

**Methods:**

Overall, 81 patients with PSC in a Japanese PSC registry recruited from 17 medical centers and hospitals, and 358 controls were enrolled. We retrospectively assessed anti-integrin αvβ6 autoantibodies using the Anti-integrin αvβ6 ELISA Kit and in-house ELISA.

**Results:**

Anti-Integrin αvβ6 ELISA Kit and in-house ELISA exhibited a significant correlation (*r* = 0.97, P < 0.001). Anti-integrin αvβ6 autoantibodies were detected in 67 of 81 (82.7%) patients with PSC and 20 of 358 (5.6%) controls, resulting in a sensitivity of 82.7% and specificity of 94.4% for PSC, using the anti-integrin αvβ6 ELISA Kit. When focusing on the presence or absence of inflammatory bowel disease (IBD), the sensitivities for PSC with ulcerative colitis, Crohn’s disease, unclassified-IBD, and without IBD were 97.8% (43/44), 100% (1/1), 80.0% (8/10), and 53.8% (7/13), respectively. Antibody concentrations were significantly higher in PSC patients without IBD than in controls (P < 0.001).

**Conclusions:**

We validated that anti-integrin αvβ6 autoantibodies have high sensitivity and specificity for diagnosing PSC. This study provides further evidence that anti-integrin αvβ6 autoantibodies are a useful biomarker for diagnosing PSC.

**Supplementary Information:**

The online version contains supplementary material available at 10.1007/s00535-024-02169-w.

## Introduction

Primary sclerosing cholangitis (PSC) is characterized by irregularly distributed bile duct fibrosis, leading to beading and stricture formation in the intrahepatic, extrahepatic, or both bile ducts. Chronic cholestasis and progressive liver dysfunction often result in end-stage cirrhosis, necessitating liver transplantation. The incidence and prevalence of PSC range from 0 to 1.3 per 100,000 individuals per year and 0 to 16.2 per 100,000 inhabitants, respectively [1]. The diagnosis of PSC is based on cholangiographic features, histology, and exclusion of other chronic cholestatic liver diseases. There are no specific findings for PSC diagnosis, and thus, some cases have the potential to be misdiagnosed as other biliary diseases. Therefore, specific biomarkers for PSC are required, although no such biomarkers have been identified to date.

Biliary epithelial cells are the main target of PSC. However, the precise pathophysiology of PSC is unknown [2]. There is a strong association between PSC and inflammatory bowel disease (IBD). Notably, 60–80% of patients with PSC have IBD in Northern Europe and the United States [3]. Our previous study indicated the presence of anti-integrin αvβ6 autoantibodies with high sensitivity and specificity in patients with ulcerative colitis (UC) and PSC [4–6]. Anti-integrin αvβ6 autoantibodies were found to be positive in 97.2% of PSC patients with IBD and 73.7% of those without IBD [6].

Integrin αvβ6 is a heterodimer of αv and β6 integrin subunits and is expressed on epithelial cells, including the biliary duct [6]. Integrin αvβ6 binds to extracellular matrix proteins such as fibronectin, tenascin-C, and vitronectin and mediates cell adhesion [7]. Integrin αvβ6 is considered to maintain the epithelial barrier function [8].

Our previous screening study was conducted on patients from only two institutions using our conventional in-house enzyme-linked immunosorbent assay (ELISA) method. This conventional ELISA method lacks quantitative capability because of its dependency on measurement conditions affecting optical density (OD). Quantification of the antibody is necessary for comparisons across various facilities without variations across different measurements. Therefore, validation studies in multiple centers are required to eliminate selection bias, and a universal method for antibody measurements needs to be developed to compare data from different facilities. For this purpose, we developed the Anti-integrin αvβ6 ELISA Kit to quantify anti-integrin αvβ6 autoantibodies.

The Intractable Hepato-Biliary Diseases Study Group in Japan has conducted the nationwide PSC registry study “Establishment of disease registry of primary sclerosing cholangitis for investigation of clinical features, natural history, and prognostic factors.” This registry aims to prospectively collect clinical information and serum samples from patients with PSC since 2019. Using these resources, in this Japanese nationwide validation study, we aimed to examine the diagnostic value of anti-integrin αvβ6 autoantibodies for PSC diagnosis.

## Methods

### Study population

This study enrolled 81 patients with PSC and 358 controls. Patients with PSC were recruited from 17 medical centers and hospitals across Japan through a nationwide PSC registry. The clinical characteristics of the patients with PSC and the controls are summarized in Table 1 and Supplemental Table 1.

**Table 1:** Characteristics of the study population

	PSC (n = 81)	CCA (n = 117)	PBC (n = 110)	AIH (n = 25)	IgG4-SC (n = 95)	SSC (n = 11)
Age, years^*^	28.4 ± 20.1	70.0 ± 8.3	64.0 ± 11.9	63.9 ± 13.3	66.6 ± 9.8	68.1 ± 12.8
Sex, *n*						
Male	52	79	14	3	75	6
Female	24	38	96	22	20	5
Unknown	5					
ALP (U/L)^†^	99.4	122.5	115.9	146.3	N/A	257
	(45.2–173.6)	(76.0–209.0)	(86.6–157.4)	(111.0–181.0)		(146.7–664.0)
T-bil (mg/dL)^†^	1.0	0.7	0.7	1.0	N/A	1.0
	(0.5–2.0)	(0.6–0.9)	(0.5–0.9)	(0.8–1.8)		(0.6–1.2)
CRP (mg/dL)^†^	0.1	0.3	0.17	0.18	N/A	1.6
	(0.03–0.50)	(0.10–0.80)	(0.08–0.57)	(0.08–0.35)		(0.5–2.1)
AST (IU/L)^†^	47.5	32	30	265	N/A	32
	(27–108)	(23–44)	(23–42)	(151–499)		(22–74)
ALT (IU/L)^†^	49	29	24	317	N/A	76
	(28–97)	(20–51)	(16–39)	(186–688)		(28.5–91)

*PSC* primary sclerosing cholangitis, *CCA* cholangiocarcinoma, *PBC* primary biliary cholangitis, *AIH* autoimmune hepatitis, *IgG4-SC* immunoglobulin G4-related sclerosing cholangitis, *SSC* secondary sclerosing cholangitis, *ALP* alkaline phosphatase, *T-bil* total bilirubin, *CRP* C-reactive protein, *AST* aspartate aminotransferase, *ALT* alanine aminotransferase, *N/A* not available

^*^Plus–minus values are presented as means ± SD

^†^Median (25th–75th percentile)

PSC was diagnosed using Japanese diagnostic criteria based on serum biochemistry, cholangiography, histological findings of the liver, association with IBD, and absence of alternative diagnoses [9]. Colonoscopy was performed on 68 of 81 patients with PSC. Among them, 44 cases of UC, 1 case of Crohn’s disease (CD), 10 cases of unclassified-IBD (IBD-U), and 13 cases with a normal colon were confirmed. As a control group, pathologically confirmed patients with cholangiocarcinoma (CCA) were recruited from the Clinical Bio-Resource Center in Kyoto University Hospital. Patients with IgG4-SC, diagnosed based on the Japanese clinical diagnostic criteria [10], were recruited from the study “IgG4-related disease in the Japanese population: a genome-wide association study”[11] Patients with autoimmune hepatitis (AIH) [12] and primary biliary cholangitis (PBC)[13] were recruited from the study “Survey of liver diseases in Ehime prefecture,” conducted at the Department of Gastroenterology and Metabology, Ehime University Graduate School of Medicine, or the “Serum cytokeratin 18 as a nexus between elevated liver and cholestatic enzymes in primary biliary cirrhosis: A surrogate of disease activity and treatment response,” conducted at the Clinical Research Center, NHO Nagasaki Medical Center. Patients with secondary sclerosing cholangitis (SSC) [14] [15] were recruited at Kyoto University Hospital. In total, 17.9% (64/358) of patients in the control groups had undergone colonoscopy. Two patients with CCA had IBD-U, whereas the others did not have IBD. Both CCA patients with IBD-U had also undergone hepatectomy, and PSC was histologically excluded.

### Analysis of cholangiographic findings

The cholangiographic findings were classified into five typical PSC-specific features: “band-like stricture,” “pruned-tree appearance,” “beaded-appearance,” “diverticulum-like outpouching,” and “shaggy appearance” [9]. The presence or absence of these findings in each patient was scored as 1 or 0, respectively, and the total score was calculated (Supplemental Table 2).

**Table 2:** Analysis for predicting antibody concentration in patients with PSC

Variable	Univariate			Multivariate		
	Regression coefficients	95% CI	P-value	Regression coefficients	95% CI	P-value
Age	−0.0277	−0.0503 to −0.00509	< 0.05	−0.00800	−0.0351 to 0.0191	0.557
T−bil	−0.0403	−0.124 to 0.0439	0.343			
ALP	−0.000148	−0.00112 to 0.000828	0.763			
CRP	−0.241	−0.552 to 0.0701	0.126			
ALT	−0.000148	−0.00237 to 0.0104	0.213			
UC	1.82	0.858 to 2.78	< 0.001	1.74	0.656 to 2.83	< 0.01

*PSC* primary sclerosing cholangitis, *T-bil* total bilirubin, *ALP* alkaline phosphatase, *CRP* C-reactive protein, *ALT* alanine aminotransferase, *UC* ulcerative colitis

### Ethical considerations

The experiments were performed in accordance with the ethical guidelines of the 1975 Declaration of Helsinki and were approved by the Ethics Committee of Kyoto University Graduate School and Faculty of Medicine (protocol number: R3344). Informed consent was obtained in the form of opt-out on the website.

### ELISA

We collaborated with the Medical and Biological Laboratories Co., Ltd. to develop the Anti-Integrin αvβ6 ELISA Kit (catalog no. 5288, Medical and Biological Laboratories, Nagoya, Japan.). The procedure for using this ELISA kit was as follows. Serum samples were diluted 1:100 by adding a reaction buffer. The standard material (recombinant monoclonal human anti-integrin αvβ6 antibody) was serially diluted with the reaction buffer from 200.0 U/mL to 0.781 U/mL. The reaction buffer served as the zero standard. ELISA plates were incubated for 60 min at room temperature with 100 μL of diluted serum or standard material. After removing the solutions in the wells and washing, 100 μL of horseradish peroxidase (HRP)-conjugated antibody was added to each well, and the plates were incubated for 60 min at room temperature. After another washing, 100 μL of substrate reagent was added to each well and incubated at room temperature for 20 min. At 450 nm, absorbance was measured following the addition of 100 μL of stop solution. The antibody concentration of each sample was determined using a calibration curve generated based on the OD value of the standard material through a 4-parameter logistic regression. Samples below the lower limit of the calibration curve were defined as having a concentration of 0 U/mL.

Our in-house ELISA method was performed as previously described [4, 6]. Briefly, microtiter plates were coated with 100 μL of 2 μg/mL of the human recombinant integrin αvβ6 protein, incubated overnight at 4 °C, blocked, and incubated with 100 µL of diluted serum (1:100) from patients for 60 min at room temperature. After washing, the plates were incubated with 100 µL of HRP-conjugated antibody at room temperature for 60 min. Following another washing step, the bound reactants were detected by incubation with 3,3′,5,5′-tetramethylbenzidine for 7 min at room temperature. The absorbance was measured at 450 nm. This in-house ELISA was performed in the presence of 1 mmol/L of MgCl_2_ and CaCl_2_ [4, 6]. The ELISA procedures were performed by two experimenters, and the adopted result was the average value derived from their efforts.

### Statistical analysis

Statistical analyses were performed using GraphPad Prism version 9.5.1 (GraphPad, La Jolla, California, USA) or R version 3.6.3. Statistical differences were assessed using the Mann–Whitney U test or the Kruskal–Wallis test for continuous data. The Dunn test was used as a post hoc test for the Kruskal–Wallis test. Spearman correlation was used to assess the correlation between antibody concentrations and clinical data and between the Anti-Integrin αvβ6 ELISA Kit and the in-house conventional ELISA method. The cut-off value was determined using Youden’s index, which is the maximum value of (sensitivity + specificity–1) derived from the receiver operating characteristics (ROC) curve. The ROCs were compared using the DeLong method. Univariate and multivariable analyses were performed using a linear regression model. Statistical significance was defined as P < 0.05.

## Results

### Comparison of the new ELISA Kit with the conventional in-house ELISA

**Fig. 1:**
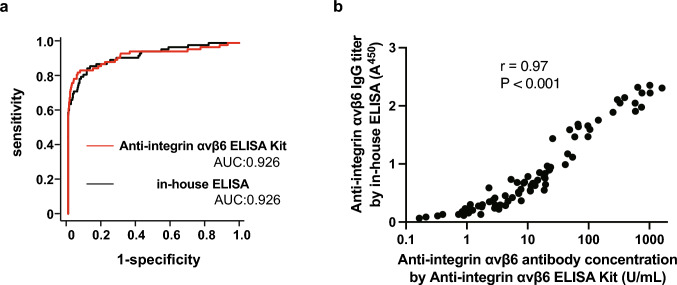
Comparing the diagnostic accuracy between the anti-integrin αvβ6 ELISA kit and the in-house conventional ELISA method. **a** ROC curves for the diagnosis of PSC obtained from the Anti-Integrin αvβ6 ELISA Kit (red line) and in-house ELISA method (black line) are shown. No significant differences were observed between the ROC curves. **b** A scatter plot shows the concentrations of anti-integrin αvβ6 autoantibodies from the Anti-Integrin αvβ6 ELISA Kit and OD values of the antibody derived from in-house conventional ELISA. Data from both methods showed a significantly strong correlation. *ELISA* enzyme-linked immunosorbent assay, *OD* optical density, *ROC* receiver operating characteristic, *PSC* primary sclerosing cholangitis

We evaluated serum samples collected from 81 patients with PSC and 358 controls. Using the Anti-Integrin αvβ6 ELISA Kit, the cut-off antibody concentration was 1.69 U/mL. The cut-off OD value of the in-house ELISA was 0.231 A^450^. The area under the ROC curve was 0.926 (95% confidence interval [CI], 0.884–0.968; P < 0.0001) for the Anti-Integrin αvβ6 ELISA Kit and 0.926 (95% confidence interval, 0.889–0.963; P < 0.0001) for our conventional in-house ELISA method. There was no significant difference between the ROC curves (P = 0.99; Fig. 1a). There was a significant correlation between anti-integrin αvβ6 autoantibody concentrations measured using the Anti-Integrin αvβ6 ELISA Kit and OD values of the antibody measured using the in-house ELISA (r = 0.97, P < 0.001; Fig. 1b).

### Anti-integrin αvβ6 autoantibodies in PSC

**Fig. 2:**
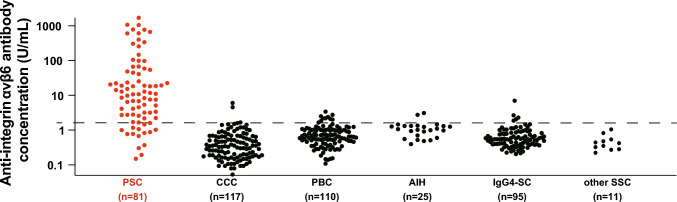
Anti-integrin αvβ6 autoantibodies in patients with PSC. Anti-integrin αvβ6 autoantibodies were quantified using the Anti-Integrin αvβ6 ELISA Kit. The cut-off value, determined by the ROC curve, is indicated by a dashed line. In total, 81 patients with PSC, 117 with CCA, 110 with PBC, 25 with AIH, 95 with IgG4-SC, and 11 with other SSC were examined, resulting in a sensitivity of 82.7% and specificity of 94.4%. *ELISA* enzyme-linked immunosorbent assay, *PSC* primary sclerosing cholangitis, *CCA* cholangiocarcinoma, *PBC* primary biliary cholangitis, *AIH* autoimmune hepatitis, *IgG4-SC* immunoglobulin G4-related sclerosing cholangitis, *SSC* secondary sclerosing cholangitis, *ROC* receiver operating characteristic

Using the Anti-Integrin αvβ6 ELISA Kit, the presence of anti-integrin αvβ6 autoantibodies was observed in 67 of 81 patients with PSC, 3 of 117 patients with CCA, 10 of 110 patients with PBC, 2 of 25 patients with AIH, 5 of 95 patients with IgG4-SC, and 0 of 11 with patients with other SSC, demonstrating a sensitivity of 82.7% and a specificity of 94.4% for the diagnosis of PSC (Fig. 2). The positive and negative predictive values were 77.0% and 96.0%, respectively.

### Anti-integrin αvβ6 autoantibodies in PSC with or without IBD

**Fig. 3:**
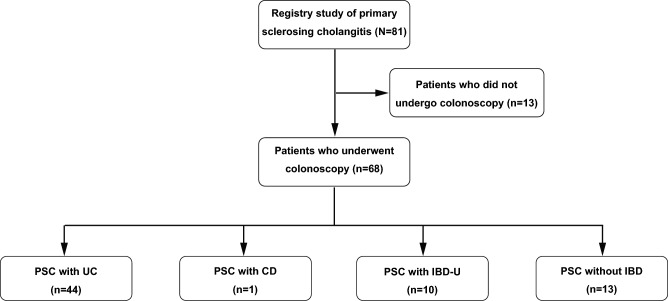
Flowchart for exploring IBD co-existence in the Japanese PSC nationwide registry study. *UC* ulcerative colitis, *CD* Crohn’s disease, *IBD-U* unclassified-inflammatory bowel disease, *PSC* primary sclerosing cholangitis

**Fig. 4:**
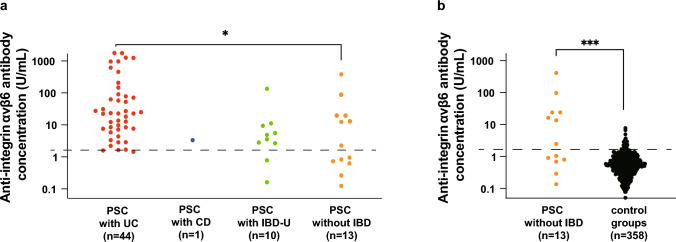
Anti-integrin αvβ6 autoantibodies in PSC with or without IBD. **a** Antibody concentrations were compared in PSC patients with or without IBD. The sensitivities of PSC patients with UC, CD, and IBD-U and those without IBD were 97.8% (43/44), 100% (1/1), 80.0% (8/10), and 53.8% (7/13), respectively. The antibody concentrations observed in PSC patients with UC (n = 44) were significantly higher than those in PSC patients without IBD (n = 13). **b** Antibody concentrations in PSC patients without IBD (n = 13) were compared with those in controls (n = 358). Anti-integrin αvβ6 antibody concentrations in PSC patients without IBD were also significantly higher than those in controls. *UC* ulcerative colitis, *CD* Crohn’s disease, *IBD-U* unclassified-inflammatory bowel disease; * P < 0.05; *** P < 0.001

Subsequently, we focused on the presence or absence of UC, CD, and IBD-U to determine whether the co-existence of IBD and PSC influenced sensitivity. Sensitivity was examined in 68 patients with PSC who underwent colonoscopy (Fig. 3). The sensitivity was 97.8% (43 of 44) in PSC with UC, 100% (1 of 1) in PSC with CD, 80.0% (8 of 10) in PSC with IBD-U, and 53.8% (7 of 13) in PSC without IBD (Fig. 4a). Autoantibody concentrations in PSC with UC were significantly higher than those in PSC without IBD (P = 0.045). On the other hand, the autoantibody concentrations in PSC patients without IBD were also significantly higher than those in controls (P < 0.001) (Fig. 4b). We compared five PSC-specific cholangiographic features—“band-like stricture” “pruned-tree appearance,” “beaded-appearance,” “diverticulum-like outpouching,” and “shaggy appearance”—and laboratory data between integrin αvβ6 autoantibody-positive and -negative PSC patients without IBD. However, we observed no significant differences in any of these features (Supplemental Table 2).

### Changes in anti-integrin αvβ6 autoantibodies throughout the clinical course

We measured the autoantibody concentrations in 37 cases with PSC who had undergone colonoscopy and had yearly follow-up blood samples. Yearly autoantibody concentrations varied within the same patient. For example, among 27 PSC patients with UC who tested positive for anti-integrin αvβ6 antibodies at enrollment, three patients tested negative at the 1-year follow-up. In six PSC patients with IBD-U, the pattern of antibody concentrations varied throughout the clinical course. Specifically, among two PSC patients with IBD-U who had positive antibody concentrations at enrollment, the antibody concentrations dropped below the cut-off point at the 1-year follow-up, but increased again above the cut-off at the 2-year follow-up in one patient. Moreover, two out of the four PSC patients without IBD initially tested negative for antibodies at enrollment, but all four tested positive at the 1-year follow-up (Supplemental Fig. 1). Notably, none of these four PSC patients without IBD developed IBD during the follow-up period.

### Relevance of anti-integrin αvβ6 autoantibodies with laboratory and clinical findings

**Fig. 5:**
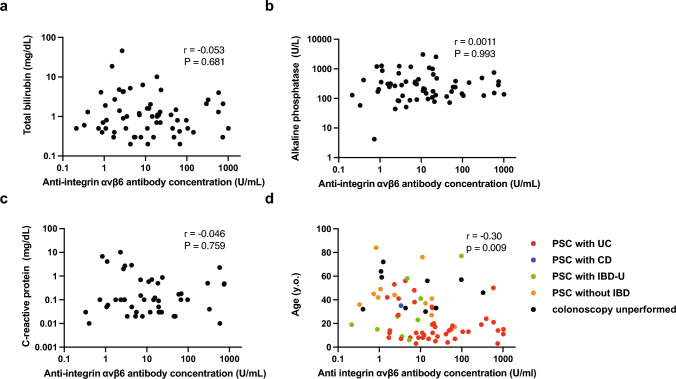
Relevance of anti-integrin αvβ6 autoantibodies with laboratory and clinical findings in PSC. We investigated the relevance of anti-integrin αvβ6 autoantibodies with laboratory and clinical findings in PSC. No significant correlation was found between the concentrations of anti-integrin αvβ6 autoantibodies and the levels of **a** total bilirubin, **b** alkaline phosphatase, and **c** C-reactive protein. **d** There was a weak negative correlation between autoantibodies and age in patients with PSC. Colored dots represent the types of accompanying IBD based on colonoscopy findings. *PSC* primary sclerosing cholangitis, *UC* ulcerative colitis, *CD* Crohn’s disease, *IBD-U* unclassified-inflammatory bowel disease

We explored the potential correlation between autoantibody concentrations and various factors such as total bilirubin, alkaline phosphatase, C-reactive protein, and age in patients with PSC. We found no significant correlation between the concentrations of anti-integrin αvβ6 autoantibodies and the levels of total bilirubin, alkaline phosphatase, and C-reactive protein (Fig. 5a–c). There was a weak negative correlation between autoantibody concentrations and age in patients with PSC (Fig. 5d). However, multivariate analysis indicated that age does not influence antibody concentration, whereas the presence of UC is a contributing factor (Table 2).

## Discussion

We previously reported that the sensitivity and specificity of anti-integrin αvβ6 antibodies for diagnosing PSC are 89.1% and 96.7%, respectively [6]. However, patients with PSC were recruited from only two institutions. The present study included all cases from the Japanese nationwide PSC registry study. We obtained results similar to those of the previous study, confirming that anti-integrin αvβ6 autoantibodies are useful biomarkers for PSC.

There are no specific biomarkers for PSC, and its diagnosis mainly depends on cholangiography. Thus, it is sometimes difficult to distinguish PSC from other hepatobiliary diseases such as CCA, IgG4-SC, AIH, or PBC [16–19]. Regarding autoantibodies, the perinuclear anti-neutrophil cytoplasmic antibody is reported to be positive in 26–94% of PSC, 22–88% of AIH, and 0–33% of PBC cases [20]. Anti-glycoprotein 2 antibody is reported to be positive in 30.8–52.2% of patients with PSC [21] and 36% of patients with CCA [22]. Radiological images of PSC also overlap with those of other biliary diseases such as CCA or IgG4-SC [23]. Although current European and American guidelines advocate magnetic resonance cholangiopancreatography as the preferred method over endoscopic retrograde cholangiopancreatography (ERCP) [17, 18, 24], some cases require ERCP to detect small or subtle biliary lesions [25, 26]. However, ERCP occasionally leads to severe complications such as fatal pancreatitis [27]. Anti-integrin αvβ6 autoantibodies could noninvasively contribute to diagnosing PSC accurately.

The genetic predisposition for PSC is shared with that of IBD [28–30], and 60–80% of patients with PSC have IBD in Northern Europe and the United States [3]. In the present study, 80.9% of patients with PSC had concomitant IBD, and 64.7% of patients with PSC had UC. The antibody concentrations in PSC patients with UC were significantly higher than those in PSC patients without IBD. These data suggest that the presence of the antibody in patients with PSC merely reflects accompanying UC. However, we found that more than half of the patients without IBD also had this antibody, and its concentrations in PSC patients without IBD were significantly higher than those in the controls. Notably, although two PSC patients without IBD who initially tested negative for anti-integrin αvβ6 antibodies at enrollment tested positive at the 1-year follow-up, they did not develop IBD during the follow-up period. Thus, the presence of this antibody indicates that it is associated with PSC as well as that of UC.

Regarding the negative correlation between autoantibody concentrations and age in patients with PSC, we speculate that accompanying IBD might have confounded the result. In this PSC registry, younger patients had IBD, especially UC. As indicated in the subgroup analysis in Fig. 4a, PSC patients with UC had higher antibody concentrations than PSC patients without IBD. Multivariate analysis revealed that the age of patients did not influence the antibody concentration whereas the presence of UC did.

Integrin αvβ6 is reportedly expressed in the colonic epithelium and bile duct epithelium, and IgGs from patients with PSC and UC exhibit inhibitory effects on the binding between integrin αvβ6 and fibronectin in vitro [4, 6]. Weil et al. reported a case of *ITGB6* homozygous germline mutation, which showed lethal cholestatic liver injuries and bloody diarrhea, clinical features of PSC and UC, respectively [31]. Taken together, anti-integrin αvβ6 autoantibodies may play an important role in the clinical manifestation of PSC as well as that of UC.

In this study, we collaborated with Medical and Biological Laboratories Co., Ltd. to establish the Anti-Integrin αvβ6 ELISA Kit, which enables easier detection of anti-integrin αvβ6 antibodies. This ELISA kit includes antigen pre-coated strip plates, rendering it ready-to-use and eliminating the need for electrolyte addition to the buffer [4]. Furthermore, standard material facilitated the standardization of anti-integrin αvβ6 autoantibody titers. Notably, the results of this kit and those of the in-house method showed a significant correlation, and there was no significant difference between the two methods in the ROC curves for the diagnosis of PSC.

This study had some limitations. Although all patients were recruited from the Japanese nationwide PSC registry, the number of patients with PSC was limited. In addition, in this PSC registry, the ages of the patients were younger and the IBD prevalence was higher than those previously reported in a nationwide survey in Japan [32], potentially limiting the generalizability of our findings. Thus, large-scale studies involving other ethnicities are warranted. Moreover, only a few patients in the control groups underwent colonoscopy. Although no abdominal pain or diarrhea was reported in patients in any of the control groups, the possibility of undiagnosed IBD cannot be ruled out. Furthermore, whether anti-integrin αvβ6 autoantibodies have pathogenic roles in PSC manifestation or a secondary phenomenon was not elucidated. Nevertheless, our findings further support evidence that anti-integrin αvβ6 autoantibodies are a useful biomarker for diagnosing PSC.

In conclusion, this study confirmed that anti-integrin αvβ6 autoantibodies had high specificity and sensitivity for diagnosing PSC in the Japanese nationwide registry with a standardized ELISA kit. These autoantibodies are useful as non-invasive diagnostic biomarkers for PSC.

## Supplementary Information

Below is the link to the electronic supplementary material.

Supplementary file1 (DOCX 391 kb)
