# Supplementary material for: Anti-integrin αvβ6 autoantibody in primary sclerosing cholangitis: a Japanese nationwide study
Source: J Gastroenterol. 2024 Nov 16;60(1):118–26. doi: 10.1007/s00535-024-02169-w (PMC11717786; doi:10.1007/s00535-024-02169-w)
Supplement: Supplementary file 1 — Supplementary file1 (DOCX 391 kb) [file 535_2024_2169_MOESM1_ESM.docx]

*Supplementary information*

**Anti-integrin αvβ6 autoantibody in primary sclerosing cholangitis: a Japanese nationwide study**

Muneji Yasuda, Masahiro Shiokawa*, Takeshi Kuwada, Yoshihiro Nishikawa, Risa Nakanishi, Ikuhisa Takimoto, Koki Chikugo, Masataka Yokode, Yuya Muramoto, Shimpei Matsumoto, Takeharu Nakamura, Sakiko Ota, Tomoaki Matsumori, Keiko Kuroda, Takahisa Hachiya, Hajime Yamazaki, Norimitsu Uza, Yuzo Kodama, Tsutomu Chiba, Toshio Fujisawa, Atsumasa Komori, Masanori Abe, Izumi Yamaguchi, Fumihiko Matsuda, Hiroyuki Isayama, Atsushi Tanaka, and Hiroshi Seno on behalf of the Japan PSC Study Group (JPSCSG)

**Corresponding author:**

Masahiro Shiokawa, MD, PhD, Department of Gastroenterology and Hepatology, Kyoto University Graduate School of Medicine, Kyoto, Japan. Tel.: +81-75-751-4319; Fax: +81-75-751-4303; Email: machan@kuhp.kyoto-u.ac.jp

**Supplemental Figures**

 **Supplemental Fig. 1 Changes in anti-integrin αvβ6 autoantibodies throughout the clinical course**

Anti-integrin αvβ6 autoantibody concentrations at the yearly follow-up are grouped by the type of accompanying IBD. **(a)** PSC with UC **(b),** PSC with IBD-U, or **(c)** PSC without IBD.

Blue lines indicate that positive antibody concentrations have dropped below the cut-off point. Red lines indicated that negative antibody concentrations increased above the cut-off.

PSC, primary sclerosing cholangitis; UC, ulcerative colitis; IBD-U, unclassified-inflammatory bowel disease

**Supplemental tables**

| Supplemental Table 1. Clinical information for primary sclerosing cholangitis patients and disease controls | | | | | | | | | | | | | |
| --- | --- | --- | --- | --- | --- | --- | --- | --- | --- | --- | --- | --- | --- |
| Sample | Age (years) | Sex | ALP (U/L) | T-bil (mg/dL) | CRP (mg/dL) | AST (IU/L) | ALT (IU/L) | PSC subtype | IBD※ | Anti-integrin αvβ6 antibody concentration (U/mL) by the Anti-Integrin αvβ6 ELISA Kit | Anti-integrin αvβ6 IgG titer (A450) by in-house ELISA | UDCA (mg/day) | Bezafibrate (mg/day) |
| PSC 01 | 36 | F | 20 | 0.6 | 0.03 | 18 | 12 | intra/extra | none | 0.33 | 0.11 | 300 | N/A |
| PSC 02 | 17 | F | 84 | 0.42 | 0.1 | 85 | 134 | intra/extra | none | 66.79 | 1.68 | 600 | N/A |
| PSC 03 | 33 | F | 174 | 1 | 0.09 | 38 | 52 | intra/extra | UC | 19.31 | 0.84 | 600 | N/A |
| PSC 04 | N/A | N/A | N/A | N/A | N/A | N/A | N/A | unknown | none | 0.17 | 0.07 | N/A | N/A |
| PSC 05 | 57 | F | 51 | 0.8 | 0.1 | 30 | 28 | intra/extra | N/A | 97.11 | 1.66 | N/A | N/A |
| PSC 06 | 27 | M | 247 | 4.2 | 0.1 | 85 | 94 | intra/extra | IBD-U | 2.86 | 0.24 | 900 | 400 |
| PSC 07 | N/A | N/A | N/A | N/A | N/A | N/A | N/A | unknown | UC | 6.70 | 0.68 | N/A | N/A |
| PSC 08 | 45 | M | 147 | 0.5 | 0.1 | 27 | 39 | intra/extra | none | 0.73 | 0.13 | N/A | N/A |
| PSC 09 | N/A | N/A | N/A | N/A | N/A | N/A | N/A | unknown | N/A | 643.60 | 2.32 | N/A | N/A |
| PSC 10 | N/A | N/A | N/A | N/A | N/A | N/A | N/A | unknown | N/A | 1611.90 | 2.30 | N/A | N/A |
| PSC 11 | 5 | M | 886 | 0.7 | 0.03 | 195 | 284 | intra/extra | UC | 22.91 | 0.90 | 600 | N/A |
| PSC 12 | 72 | M | N/A | N/A | N/A | N/A | N/A | intra/extra | N/A | 1.25 | 0.20 | 300 | N/A |
| PSC 13 | 49 | M | 125 | 1.9 | 0.05 | 21 | 33 | intra/extra | none | 1.07 | 0.16 | 600 | N/A |
| PSC 14 | N/A | N/A | N/A | N/A | N/A | N/A | N/A | unknown | N/A | 0.83 | 0.17 | N/A | N/A |
| PSC 15 | 32 | M | 146 | 1.3 | 0.01 | 52 | 109 | unknown | N/A | 0.40 | 0.13 | 600 | N/A |
| PSC 16 | 8 | M | 76 | 0.2 | N/A | 23 | 11 | intra | UC | 11.35 | 0.63 | 750 | N/A |
| PSC 17 | 14 | F | 25 | 0.3 | 0.02 | 14 | 7 | intra | UC | 48.82 | 1.59 | 900 | N/A |
| PSC 18 | 8 | M | 86 | 0.3 | N/A | 27 | 14 | extra | UC | 1.70 | 0.13 | 200 | N/A |
| PSC 19 | 8 | M | 408 | 0.3 | 0.03 | 165 | 327 | intra/extra | UC | 5.59 | 0.42 | 450 | N/A |
| PSC 20 | 12 | M | 1073 | 1.6 | N/A | 139 | 213 | intra | UC | 11.02 | 0.55 | 600 | N/A |
| PSC 21 | 7 | M | 380 | 0.3 | 0.12 | 58 | 63 | intra/extra | UC | 15.68 | 0.85 | 510 | N/A |
| PSC 22 | 14 | M | 110 | 2.7 | N/A | 167 | 251 | intra/extra | UC | 1.80 | 0.30 | 900 | N/A |
| PSC 23 | 19 | M | N/A | N/A | 0.05 | 23 | 29 | intra | UC | 19.29 | 0.75 | 600 | N/A |
| PSC 24 | 56 | M | 129 | 5.2 | 2.9 | 91 | 28 | intra/extra | UC | 4.28 | 0.45 | 600 | 400 |
| PSC 25 | 41 | M | 429 | 4.3 | 2.7 | 127 | 61 | intra/extra | UC | 2.93 | 0.29 | N/A | 400 |
| PSC 26 | 26 | F | 30 | 46.2 | 2 | 145 | 91 | intra | IBD-U | 2.72 | 0.38 | N/A | N/A |
| PSC 27 | 7 | F | 134 | 0.2 | 0.18 | 37 | 36 | unknown | UC | 67.88 | 1.64 | 270 | 400 |
| PSC 28 | 3 | F | 106 | 0.3 | N/A | 59 | 66 | unknown | UC | 7.75 | 0.56 | 450 | N/A |
| PSC 29 | 13 | M | 43 | 0.5 | N/A | 17 | 15 | intra | UC | 105.12 | 1.59 | 900 | 400 |
| PSC 30 | 17 | M | 45 | 1.3 | N/A | 52 | 45 | unknown | UC | 22.55 | 0.89 | 600 | N/A |
| PSC 31 | 4 | M | N/A | 0.5 | 0.02 | 67 | 81 | intra/extra | UC | 45.17 | 1.17 | 600 | N/A |
| PSC 32 | 18 | F | 85 | 0.8 | 0.17 | 136 | 131 | unknown | UC | 59.40 | 1.47 | 900 | N/A |
| PSC 33 | 11 | M | 41 | 1.1 | N/A | 31 | 52 | unknown | UC | 41.37 | 0.99 | 600 | N/A |
| PSC 34 | 13 | F | 120 | 0.4 | N/A | 20 | 9 | unknown | UC | 144.93 | 1.75 | N/A | 400 |
| PSC 35 | 3 | M | 99 | 0.3 | 0.45 | 39 | 40 | unknown | UC | 740.31 | 1.98 | 420 | N/A |
| PSC 36 | 11 | M | 48 | 0.5 | N/A | 15 | 14 | intra/extra | UC | 1011.70 | 2.35 | 600 | N/A |
| PSC 37 | 33 | F | 18 | 0.2 | 0.05 | 24 | 22 | unknown | N/A | 4.32 | 0.28 | 600 | N/A |
| PSC 38 | 12 | M | 32 | N/A | 0.02 | 25 | 23 | unknown | UC | 7.84 | 0.37 | 900 | 400 |
| PSC 39 | 9 | M | 43 | 1 | N/A | 31 | 39 | unknown | IBD-U | 3.54 | 0.30 | 300 | N/A |
| PSC 40 | 6 | F | 30 | 0.3 | 0.02 | 34 | 47 | intra | IBD-U | 5.32 | 0.73 | 600 | 400 |
| PSC 41 | 10 | M | 72 | 1.6 | 0.03 | 36 | 48 | intra | UC | 12.07 | 0.67 | 600 | N/A |
| PSC 42 | 14 | M | 130 | 2.1 | 0.48 | 420 | 337 | intra/extra | UC | 752.44 | 2.22 | 900 | N/A |
| PSC 43 | 15 | M | N/A | N/A | N/A | N/A | N/A | unknown | UC | 1023.79 | 2.22 | N/A | N/A |
| PSC 44 | 12 | M | 226 | 1.1 | 0.03 | 155 | 198 | intra/extra | UC | 19.66 | 0.65 | 450 | N/A |
| PSC 45 | 19 | M | 45 | 0.5 | N/A | 34 | 34 | unknown | IBD-U | 0.21 | 0.09 | 600 | N/A |
| PSC 46 | 15 | M | 60 | 0.4 | N/A | 26 | 15 | unknown | IBD-U | 0.89 | 0.11 | 900 | N/A |
| PSC 47 | 12 | F | 100 | 0.4 | 0.1 | 64 | 53 | intra | UC | 1.76 | 0.26 | 600 | N/A |
| PSC 48 | 7 | F | 99 | 0.5 | 0.02 | 30 | 14 | intra | UC | 3.35 | 0.25 | 400 | 200 |
| PSC 49 | 84 | F | 419 | 4.1 | 6.8 | 122 | 50 | intra/extra | none | 0.84 | 0.18 | 600 | N/A |
| PSC 50 | 53 | M | 93 | 4.8 | 10.1 | 139 | 193 | unknown | UC | 2.30 | 0.59 | 600 | N/A |
| PSC 51 | 77 | F | N/A | N/A | N/A | N/A | N/A | unknown | IBD-U | 97.89 | 1.47 | 600 | 400 |
| PSC 52 | 38 | M | 127 | 1.1 | 0.15 | 37 | 46 | unknown | UC | 7.15 | 0.49 | 1200 | N/A |
| PSC 53 | 19 | M | 172 | 2.1 | 0.5 | 180 | 204 | intra | UC | 297.05 | 2.10 | 300 | N/A |
| PSC 54 | 23 | M | 443 | 6.3 | 0.1 | 101 | 143 | intra | IBD-U | 8.61 | 0.67 | 900 | N/A |
| PSC 55 | 42 | M | 309 | 18.66 | N/A | 122 | 97 | intra | UC | 1.55 | 0.28 | 600 | N/A |
| PSC 56 | 48 | F | 116 | 1.2 | 0.7 | 63 | 51 | intra/extra | UC | 7.06 | 0.51 | N/A | 400 |
| PSC 57 | 56 | M | 34 | 1 | 0.1 | 19 | 14 | intra | N/A | 14.60 | 0.72 | 900 | N/A |
| PSC 58 | 27 | M | 38 | 0.7 | 0.5 | 21 | 29 | intra/extra | none | 18.58 | 0.77 | N/A | N/A |
| PSC 59 | 20 | M | 27 | 1.1 | 0.03 | 14 | 20 | intra/extra | UC | 20.91 | 0.96 | N/A | N/A |
| PSC 60 | 34 | M | 350 | 10.1 | N/A | 167 | 123 | intra/extra | UC | 18.89 | 0.77 | N/A | N/A |
| PSC 61 | 50 | M | 262 | 1.3 | 2.3 | 103 | 84 | extra | UC | 582.80 | 2.05 | N/A | N/A |
| PSC 62 | 58 | F | N/A | N/A | N/A | N/A | N/A | unknown | IBD-U | 4.74 | 0.36 | N/A | N/A |
| PSC 63 | 64 | M | 443 | 0.5 | 4.1 | 30 | 20 | intra | N/A | 1.10 | 0.24 | 600 | N/A |
| PSC 64 | 33 | M | 167 | 4.68 | 0.87 | 131 | 162 | unknown | N/A | 23.82 | 0.94 | 900 | N/A |
| PSC 65 | 30 | F | 52 | 2.03 | 0.71 | 254 | 234 | unknown | N/A | 13.19 | 0.63 | 900 | N/A |
| PSC 66 | 35 | M | N/A | N/A | N/A | N/A | N/A | unknown | CD | 3.35 | 0.22 | 900 | N/A |
| PSC 67 | 21 | M | 54 | 4.01 | 0.01 | 69 | 81 | unknown | UC | 587.63 | 1.91 | 600 | N/A |
| PSC 68 | 24 | F | N/A | N/A | N/A | N/A | N/A | unknown | UC | 391.80 | 2.14 | N/A | N/A |
| PSC 69 | 37 | F | N/A | N/A | N/A | N/A | N/A | unknown | none | 13.13 | 0.67 | 900 | N/A |
| PSC 70 | 46 | M | 44 | 2.64 | 0.04 | 58 | 57 | unknown | N/A | 328.97 | 2.05 | 900 | N/A |
| PSC 71 | 76 | F | 182 | 0.5 | 0.58 | 26 | 13 | intra/extra | none | 11.08 | 0.53 | N/A | 400 |
| PSC 72 | 15 | M | 60 | 1.48 | N/A | 81 | 98 | unknown | UC | 54.61 | 1.12 | 600 | N/A |
| PSC 73 | 41 | M | N/A | N/A | N/A | N/A | N/A | unknown | none | 18.66 | 0.55 | N/A | N/A |
| PSC 74 | 42 | M | 71 | 0.7 | N/A | 34 | 47 | intra/extra | none | 0.94 | 0.23 | 600 | 400 |
| PSC 75 | 27 | M | 29 | 0.9 | 0.1 | 49 | 70 | intra/extra | UC | 2.89 | 0.41 | 600 | 400 |
| PSC 76 | 44 | M | 15 | 1.4 | 0.1 | 36 | 33 | intra/extra | none | 2.35 | 0.35 | 600 | N/A |
| PSC 77 | N/A | M | 0 | N/A | N/A | N/A | N/A | unknown | none | 251.32 | 1.89 | N/A | N/A |
| PSC 78 | 59 | M | 304 | 0.81 | 0.06 | 46 | 46 | intra | N/A | 1.14 | 0.30 | 600 | N/A |
| PSC 79 | 9 | F | N/A | N/A | N/A | N/A | N/A | unknown | UC | 25.69 | 1.44 | N/A | N/A |
| PSC 80 | 8 | M | N/A | N/A | N/A | N/A | N/A | unknown | UC | 8.87 | 0.68 | N/A | N/A |
| PSC 81 | 41 | F | N/A | N/A | N/A | N/A | N/A | unknown | IBD-U | 10.10 | 0.78 | N/A | N/A |
| CCA 001 | 67 | F | 59 | 1.3 | 0.16 | 36 | 35 |  | N/A | 0.10 | 0.05 | 0 | 0 |
| CCA 002 | 81 | M | 98 | 0.7 | 0.48 | 39 | 74 |  | N/A | 0.21 | 0.04 | 0 | 0 |
| CCA 003 | 81 | M | 37 | 0.5 | 0.1 | 17 | 23 |  | N/A | 0.24 | 0.07 | 0 | 0 |
| CCA 004 | 69 | M | 42 | 0.5 | 0.44 | 73 | 89 |  | N/A | 0.57 | 0.08 | 0 | 0 |
| CCA 005 | 65 | M | 21 | 0.4 | 0.78 | 10 | 7 |  | N/A | 4.73 | 0.10 | 0 | 0 |
| CCA 006 | 74 | M | 35 | 0.7 | 0.1 | 29 | 24 |  | N/A | 0.18 | 0.04 | 0 | 0 |
| CCA 007 | 72 | M | 43 | 0.7 | 0.63 | 27 | 30 |  | N/A | 0.18 | 0.05 | 0 | 0 |
| CCA 008 | 72 | M | 31 | 0.7 | 0.15 | 18 | 16 |  | N/A | 1.30 | 0.13 | 0 | 0 |
| CCA 009 | 76 | M | 133 | 0.8 | 3.8 | 35 | 31 |  | N/A | 0.21 | 0.05 | 0 | 0 |
| CCA 010 | 73 | M | 30 | 0.7 | 0.8 | 24 | 7 |  | N/A | 1.28 | 0.54 | 0 | 0 |
| CCA 011 | 72 | M | 38 | 0.6 | 0.5 | 21 | 13 |  | N/A | 0.18 | 0.15 | 0 | 0 |
| CCA 012 | 78 | M | 19 | 0.5 | 0.2 | 21 | 18 |  | N/A | 0.19 | 0.03 | 0 | 0 |
| CCA 013 | 69 | M | 164 | 1 | 1.2 | 63 | 50 |  | N/A | 0.13 | 0.07 | 0 | 0 |
| CCA 014 | 59 | M | 22 | 0.5 | 1 | 40 | 56 |  | N/A | 0.10 | 0.05 | 0 | 0 |
| CCA 015 | 75 | M | 32 | 0.7 | 0.1 | 19 | 13 |  | N/A | 0.39 | 0.07 | 0 | 0 |
| CCA 016 | 51 | M | 26 | 0.8 | 0.1 | 19 | 16 |  | none | 0.15 | 0.04 | 0 | 0 |
| CCA 017 | 56 | M | 27 | 1.4 | 0.1 | 19 | 31 |  | N/A | 0.16 | 0.11 | 0 | 0 |
| CCA 018 | 67 | M | 38 | 0.4 | 0.1 | 18 | 11 |  | none | 1.07 | 0.07 | 0 | 0 |
| CCA 019 | 69 | M | 28 | 0.6 | 0.2 | 39 | 50 |  | N/A | 0.51 | 0.13 | 0 | 0 |
| CCA 020 | 70 | M | 28 | 0.5 | 0.1 | 14 | 17 |  | N/A | 0.16 | 0.08 | 0 | 0 |
| CCA 021 | 76 | M | 55 | 0.4 | 0.4 | 29 | 20 |  | none | 0.54 | 0.06 | 0 | 0 |
| CCA 022 | 73 | F | 187 | 0.6 | 0.3 | 41 | 49 |  | N/A | 0.10 | 0.03 | 0 | 0 |
| CCA 023 | 67 | M | 331 | 0.7 | 1 | 51 | 62 |  | N/A | 0.38 | 0.11 | 0 | 0 |
| CCA 024 | 70 | F | 95 | 1.1 | 0.8 | 37 | 47 |  | none | 0.41 | 0.04 | 0 | 0 |
| CCA 025 | 71 | M | 121 | 0.8 | 0.4 | 58 | 20 |  | N/A | 0.93 | 0.03 | 0 | 0 |
| CCA 026 | 69 | F | 181 | 0.8 | 0.5 | 51 | 47 |  | none | 0.55 | 0.12 | 0 | 0 |
| CCA 027 | 70 | M | 315 | 0.8 | 0.1 | 76 | 161 |  | N/A | 0.15 | 0.06 | 0 | 0 |
| CCA 028 | 70 | M | 393 | 1 | 0.5 | 35 | 42 |  | none | 0.16 | 0.07 | 0 | 0 |
| CCA 029 | 63 | M | 154 | 0.8 | 0.1 | 63 | 203 |  | N/A | 0.31 | 0.11 | 0 | 0 |
| CCA 030 | 73 | M | 338 | 0.4 | 0.4 | 46 | 58 |  | N/A | 0.25 | 0.06 | 0 | 0 |
| CCA 031 | 80 | M | 92 | 0.6 | 0.1 | 26 | 24 |  | N/A | 1.28 | 0.09 | 0 | 0 |
| CCA 032 | 68 | F | 118 | 0.8 | 0.1 | 27 | 30 |  | N/A | 0.33 | 0.08 | 0 | 0 |
| CCA 033 | 68 | F | 355 | 0.8 | 0.2 | 74 | 126 |  | N/A | 0.33 | 0.04 | 0 | 0 |
| CCA 034 | 74 | M | 68 | 0.9 | 0.1 | 23 | 28 |  | N/A | 0.33 | 0.06 | 0 | 0 |
| CCA 035 | 79 | F | 93 | 0.4 | 0.1 | 25 | 11 |  | N/A | 0.28 | 0.07 | 0 | 0 |
| CCA 036 | 73 | F | 77 | 1.6 | 0.1 | 62 | 53 |  | N/A | 0.12 | 0.04 | 0 | 0 |
| CCA 037 | 69 | M | 103 | 0.7 | 0.1 | 29 | 39 |  | none | 0.22 | 0.04 | 0 | 0 |
| CCA 038 | 67 | F | 476 | 1.2 | 0.6 | 40 | 49 |  | none | 0.52 | 0.06 | 0 | 0 |
| CCA 039 | 69 | M | 302 | 0.8 | 11.5 | 22 | 42 |  | none | 0.39 | 0.13 | 0 | 0 |
| CCA 040 | 66 | F | 173 | 0.5 | 13.6 | 16 | 11 |  | none | 0.18 | 0.10 | 0 | 0 |
| CCA 041 | 76 | F | 93 | 0.7 | 0.1 | 35 | 16 |  | N/A | 0.41 | 0.07 | 0 | 0 |
| CCA 042 | 88 | F | 217 | 1.6 | 0.2 | 31 | 25 |  | N/A | 0.24 | 0.09 | 0 | 0 |
| CCA 043 | 72 | F | 123 | 0.4 | 1.2 | 27 | 40 |  | N/A | 0.28 | 0.09 | 0 | 0 |
| CCA 044 | 84 | F | 82 | 2.2 | 19.9 | 71 | 114 |  | none | 0.06 | 0.05 | 0 | 0 |
| CCA 045 | 74 | F | 113 | 0.9 | 4.6 | 50 | 51 |  | N/A | 0.13 | 0.04 | 0 | 0 |
| CCA 046 | 69 | M | 144 | 2.2 | 0.8 | 92 | 89 |  | N/A | 0.41 | 0.08 | 0 | 0 |
| CCA 047 | 77 | M | 300 | 1.3 | 1.6 | 42 | 118 |  | N/A | 0.34 | 0.07 | 0 | 0 |
| CCA 048 | 58 | M | 58 | 0.7 | 2.4 | 87 | 68 |  | none | 0.25 | 0.06 | 0 | 0 |
| CCA 049 | 56 | M | 160 | 1.8 | 0.5 | 53 | 65 |  | N/A | 1.16 | 0.15 | 0 | 0 |
| CCA 050 | 75 | M | 180 | 0.3 | 0.4 | 23 | 22 |  | IBD-U | 0.38 | 0.05 | 0 | 0 |
| CCA 051 | 73 | M | 263 | 0.7 | 0.1 | 45 | 56 |  | N/A | 0.10 | 0.04 | 0 | 0 |
| CCA 052 | 84 | M | 119 | 0.4 | 2.3 | 17 | 14 |  | N/A | 0.36 | 0.08 | 0 | 0 |
| CCA 053 | 83 | F | 123 | 0.9 | 0.3 | 28 | 18 |  | IBD-U | 0.12 | 0.03 | 0 | 0 |
| CCA 054 | 71 | F | 55 | 0.6 | 0.3 | 35 | 28 |  | N/A | 0.18 | 0.06 | 0 | 0 |
| CCA 055 | 75 | M | 146 | 0.4 | 0.3 | 33 | 27 |  | N/A | 0.21 | 0.19 | 0 | 0 |
| CCA 056 | 63 | M | 139 | 1.2 | 1.5 | 39 | 85 |  | N/A | 6.28 | 0.44 | 0 | 0 |
| CCA 057 | 70 | M | 104 | 0.5 | 0.1 | 24 | 21 |  | none | 0.09 | 0.03 | 0 | 0 |
| CCA 058 | 64 | F | 91 | 0.5 | 6.1 | 28 | 13 |  | N/A | 0.28 | 0.07 | 0 | 0 |
| CCA 059 | 65 | M | 125 | 0.7 | 9.7 | 19 | 15 |  | N/A | 0.67 | 0.10 | 0 | 0 |
| CCA 060 | 75 | M | 127 | 0.5 | 0.1 | 26 | 24 |  | none | 0.18 | 0.06 | 0 | 0 |
| CCA 061 | 77 | F | 551 | 1.1 | 3.2 | 71 | 134 |  | none | 0.12 | 0.04 | 0 | 0 |
| CCA 062 | 76 | M | 54 | 0.6 | 0.1 | 21 | 23 |  | N/A | 0.27 | 0.12 | 0 | 0 |
| CCA 063 | 66 | M | 415 | 0.6 | 0.2 | 42 | 43 |  | N/A | 0.36 | 0.09 | 0 | 0 |
| CCA 064 | 66 | M | 72 | 0.6 | 0.5 | 23 | 16 |  | none | 0.39 | 0.06 | 0 | 0 |
| CCA 065 | 53 | M | 112 | 1.5 | 0.1 | 24 | 34 |  | N/A | 0.16 | 0.09 | 0 | 0 |
| CCA 066 | 68 | F | 223 | 0.4 | 0.1 | 21 | 25 |  | none | 0.19 | 0.07 | 0 | 0 |
| CCA 067 | 74 | M | 122 | 0.3 | 0.4 | 27 | 16 |  | none | 0.22 | 0.14 | 0 | 0 |
| CCA 068 | 81 | F | 76 | 0.7 | 0.1 | 17 | 7 |  | N/A | 0.21 | 0.05 | 0 | 0 |
| CCA 069 | 76 | M | 307 | 3.2 | 0.8 | 29 | 42 |  | none | 0.12 | 0.03 | 0 | 0 |
| CCA 070 | 80 | M | 78 | 0.6 | 0.2 | 42 | 26 |  | N/A | 0.73 | 0.07 | 0 | 0 |
| CCA 071 | 82 | F | 103 | 1.6 | 0.2 | 21 | 13 |  | N/A | 0.27 | 0.05 | 0 | 0 |
| CCA 072 | 67 | F | 186 | 0.7 | 0.4 | 50 | 28 |  | none | 0.39 | 0.04 | 0 | 0 |
| CCA 073 | 78 | M | 105 | 0.6 | 2.5 | 29 | 26 |  | none | 1.00 | 0.21 | 0 | 0 |
| CCA 074 | 66 | M | 48 | 0.8 | 0.1 | 22 | 14 |  | none | 0.31 | 0.12 | 0 | 0 |
| CCA 075 | 53 | M | 224 | 0.5 | 0.1 | 30 | 23 |  | N/A | 0.85 | 0.13 | 0 | 0 |
| CCA 076 | 68 | M | 82 | 0.8 | 0.1 | 27 | 68 |  | N/A | 0.09 | 0.07 | 0 | 0 |
| CCA 077 | 76 | M | 91 | 0.7 | 0.3 | 29 | 22 |  | none | 0.13 | 0.04 | 0 | 0 |
| CCA 078 | 52 | M | 73 | 0.6 | 0.5 | 32 | 22 |  | N/A | 0.20 | 0.07 | 0 | 0 |
| CCA 079 | 68 | M | 120 | 1 | 0.1 | 37 | 35 |  | N/A | 1.76 | 0.10 | 0 | 0 |
| CCA 080 | 66 | M | 129 | 0.7 | 0.6 | 33 | 27 |  | N/A | 0.71 | 0.21 | 0 | 0 |
| CCA 081 | 79 | F | 303 | 1.5 | 3.4 | 42 | 39 |  | none | 0.20 | 0.16 | 0 | 0 |
| CCA 082 | 68 | M | 91 | 0.6 | 0.1 | 36 | 43 |  | none | 0.19 | 0.07 | 0 | 0 |
| CCA 083 | 74 | M | 358 | 0.3 | 0.2 | 40 | 56 |  | none | 0.22 | 0.09 | 0 | 0 |
| CCA 084 | 72 | F | 149 | 0.3 | 6.8 | 16 | 10 |  | N/A | 0.31 | 0.05 | 0 | 0 |
| CCA 085 | 47 | F | 177 | 1.3 | 2.1 | 38 | 25 |  | N/A | 0.52 | 0.05 | 0 | 0 |
| CCA 086 | 73 | M | 163 | 0.9 | 0.1 | 44 | 81 |  | N/A | 0.58 | 0.14 | 0 | 0 |
| CCA 087 | 71 | M | 239 | 1 | 0.3 | 32 | 38 |  | none | 0.99 | 0.06 | 0 | 0 |
| CCA 088 | 57 | F | 238 | 0.4 | 1.6 | 24 | 29 |  | none | 1.51 | 0.20 | 0 | 0 |
| CCA 089 | 48 | F | 186 | 0.6 | 0.1 | 32 | 45 |  | N/A | 0.74 | 0.10 | 0 | 0 |
| CCA 090 | 79 | M | 79 | 0.6 | 0.1 | 22 | 14 |  | N/A | 0.72 | 0.07 | 0 | 0 |
| CCA 091 | 78 | F | 295 | 2.7 | 1.2 | 147 | 84 |  | N/A | 1.15 | 0.21 | 0 | 0 |
| CCA 092 | 83 | M | 165 | 4.6 | 1.1 | 87 | 68 |  | N/A | 1.69 | 0.21 | 0 | 0 |
| CCA 093 | 54 | M | 117 | 0.8 | 4.5 | 19 | 25 |  | none | 0.71 | 0.31 | 0 | 0 |
| CCA 094 | 71 | M | 240 | 0.6 | 0 | 57 | 60 |  | none | 0.66 | 0.08 | 0 | 0 |
| CCA 095 | 73 | F | 280 | 0.7 | 0.7 | 29 | 25 |  | N/A | 0.36 | 0.08 | 0 | 0 |
| CCA 096 | 71 | F | 312 | 0.5 | 0.2 | 35 | 46 |  | N/A | 1.15 | 0.12 | 0 | 0 |
| CCA 097 | 66 | M | 135 | 0.6 | 0.2 | 14 | 11 |  | none | 0.58 | 0.06 | 0 | 0 |
| CCA 098 | 70 | M | 200 | 0.9 | 0.2 | 83 | 123 |  | N/A | 0.50 | 0.11 | 0 | 0 |
| CCA 099 | 59 | M | 250 | 1.3 | 0.4 | 28 | 24 |  | none | 1.56 | 0.14 | 0 | 0 |
| CCA 100 | 76 | M | 294 | 0.6 | 8.3 | 23 | 12 |  | N/A | 0.42 | 0.04 | 0 | 0 |
| CCA 101 | 72 | M | 104 | 0.5 | 0.2 | 41 | 49 |  | none | 0.56 | 0.02 | 0 | 0 |
| CCA 102 | 62 | M | 194 | 0.5 | 3 | 25 | 28 |  | N/A | 1.60 | 0.24 | 0 | 0 |
| CCA 103 | 76 | F | 81 | 0.6 | 0.1 | 14 | 6 |  | N/A | 0.92 | 0.07 | 0 | 0 |
| CCA 104 | 72 | M | 136 | 1.6 | 0.1 | 44 | 52 |  | none | 0.69 | 0.11 | 0 | 0 |
| CCA 105 | 73 | F | 59 | 1.4 | 3.9 | 209 | 208 |  | N/A | 0.47 | 0.10 | 0 | 0 |
| CCA 106 | 59 | M | 64 | 0.8 | 0.2 | 22 | 22 |  | N/A | 0.69 | 0.12 | 0 | 0 |
| CCA 107 | 77 | F | 294 | 1.2 | 0.3 | 64 | 107 |  | N/A | 0.61 | 0.09 | 0 | 0 |
| CCA 108 | 65 | M | 149 | 0.3 | 0.2 | 26 | 43 |  | none | 0.49 | 0.11 | 0 | 0 |
| CCA 109 | 82 | M | 323 | 0.7 | 0.7 | 36 | 29 |  | N/A | 0.75 | 0.15 | 0 | 0 |
| CCA 110 | 67 | F | 214 | 6.4 | 2.9 | 62 | 27 |  | none | 0.51 | 0.10 | 0 | 0 |
| CCA 111 | 73 | M | 47 | 0.9 | 0.1 | 21 | 16 |  | none | 0.75 | 0.05 | 0 | 0 |
| CCA 112 | 63 | F | 35 | 0.9 | 3.7 | 218 | 205 |  | none | 0.83 | 0.08 | 0 | 0 |
| CCA 113 | 77 | M | 193 | 0.7 | 0.3 | 46 | 43 |  | N/A | 0.74 | 0.06 | 0 | 0 |
| CCA 114 | 77 | F | 149 | 0.7 | 0.7 | 18 | 20 |  | none | 0.47 | 0.09 | 0 | 0 |
| CCA 115 | 67 | M | 312 | 0.8 | 0.6 | 40 | 42 |  | N/A | 0.88 | 0.14 | 0 | 0 |
| CCA 116 | 65 | M | 98 | 0.7 | 0.1 | 31 | 22 |  | none | 0.43 | 0.06 | 0 | 0 |
| CCA 117 | 41 | F | 209 | 0.9 | 0.1 | 63 | 67 |  | N/A | 1.50 | 0.14 | 0 | 0 |
| PBC 001 | 57 | M | 168 | 1.1 | N/A | 15 | 11 |  | N/A | 1.22 | 0.26 | 600 | 0 |
| PBC 002 | 78 | F | 280 | 1 | N/A | 23 | 12 |  | none | 1.37 | 0.29 | 600 | 0 |
| PBC 003 | 68 | F | 81 | 2 | <0.30 | 23 | 9 |  | N/A | 1.03 | 0.30 | 600 | 0 |
| PBC 004 | 58 | F | 163 | 0.7 | N/A | 19 | 13 |  | N/A | 1.08 | 0.30 | 600 | 0 |
| PBC 005 | 84 | F | 163 | 0.7 | <0.30 | 25 | 15 |  | N/A | 2.29 | 0.33 | 600 | 0 |
| PBC 006 | 80 | F | 180 | 0.9 | N/A | 50 | 27 |  | N/A | 1.12 | 0.27 | 600 | 0 |
| PBC 007 | 61 | F | 113 | 0.4 | <0.30 | 21 | 13 |  | N/A | 1.08 | 0.16 | 600 | 0 |
| PBC 008 | 57 | F | 153 | 0.5 | N/A | 44 | 39 |  | N/A | 0.45 | 0.15 | 600 | 0 |
| PBC 009 | 44 | F | 428 | 6.9 | <0.30 | 109 | 104 |  | none | 0.82 | 0.23 | 1200 | 0 |
| PBC 010 | 66 | F | 148 | 0.9 | N/A | 23 | 15 |  | none | 0.63 | 0.21 | 900 | 0 |
| PBC 011 | 78 | F | 260 | 1 | 0.56 | 76 | 68 |  | none | 0.81 | 0.29 | 300 | 0 |
| PBC 012 | 63 | M | 248 | 0.5 | 0.8 | 43 | 22 |  | N/A | 1.37 | 0.20 | 600 | 0 |
| PBC 013 | 57 | M | 122 | 1.1 | N/A | 16 | 16 |  | N/A | 0.87 | 0.18 | 900 | 0 |
| PBC 014 | 73 | F | 95 | 0.5 | N/A | 26 | 25 |  | N/A | 0.61 | 0.14 | 600 | 0 |
| PBC 015 | 77 | F | 75 | 0.5 | N/A | 26 | 16 |  | N/A | 1.06 | 0.13 | 600 | 400 |
| PBC 016 | 77 | M | 136 | 2.7 | N/A | 96 | 51 |  | none | 2.52 | 0.36 | 900 | 400 |
| PBC 017 | 59 | F | 300 | 7.2 | 0.89 | 244 | 129 |  | none | 2.22 | 0.35 | 900 | 200 |
| PBC 018 | 84 | F | 96 | 2.4 | N/A | 53 | 36 |  | N/A | 1.22 | 0.52 | 900 | 200 |
| PBC 019 | 78 | F | 96 | 1.3 | N/A | 19 | 9 |  | N/A | 1.02 | 0.19 | 600 | 0 |
| PBC 020 | 78 | F | 75 | 0.4 | N/A | 37 | 25 |  | N/A | 0.70 | 0.18 | 600 | 400 |
| PBC 021 | 81 | F | 57 | 0.6 | 0.6 | 19 | 9 |  | N/A | 2.90 | 0.39 | 600 | 0 |
| PBC 022 | 83 | F | 71 | 0.6 | N/A | 36 | 16 |  | N/A | 0.77 | 0.21 | 600 | 0 |
| PBC 023 | 76 | M | 114 | 0.5 | <0.30 | 37 | 28 |  | none | 0.57 | 0.25 | 900 | 200 |
| PBC 024 | 89 | F | 109 | 0.6 | N/A | 35 | 20 |  | none | 0.38 | 0.12 | 600 | 400 |
| PBC 025 | 75 | F | 54 | 0.7 | 0.16 | 20 | 16 |  | N/A | 0.31 | 0.15 | 600 | 0 |
| PBC 026 | 49 | M | 103 | 0.8 | <0.14 | 29 | 21 |  | N/A | 0.45 | 0.14 | 600 | 0 |
| PBC 027 | 72 | F | 118 | 1.2 | N/A | 27 | 22 |  | N/A | 0.31 | 0.13 | 600 | 0 |
| PBC 028 | 51 | F | 143 | 0.5 | N/A | 61 | 61 |  | N/A | 0.77 | 0.17 | 900 | 400 |
| PBC 029 | 75 | F | 79 | 1.1 | N/A | 22 | 11 |  | N/A | 0.59 | 0.18 | 300 | 0 |
| PBC 030 | 69 | M | 145 | 1 | N/A | 41 | 31 |  | none | 0.73 | 0.18 | 600 | 0 |
| PBC 031 | 72 | F | 86 | 0.6 | N/A | 31 | 31 |  | N/A | 0.70 | 0.14 | 600 | 0 |
| PBC 032 | 70 | F | 98 | 1 | N/A | 24 | 19 |  | N/A | 1.10 | 0.23 | 0 | 0 |
| PBC 033 | 80 | F | 102 | 0.4 | N/A | 23 | 10 |  | none | 2.32 | 0.34 | 600 | 0 |
| PBC 034 | 70 | F | 131 | 0.6 | N/A | 27 | 17 |  | N/A | 0.73 | 0.23 | 600 | 0 |
| PBC 035 | 81 | F | 72 | 1 | N/A | 19 | 10 |  | N/A | 1.18 | 0.26 | 600 | 0 |
| PBC 036 | 64 | F | 106 | 0.5 | N/A | 23 | 6 |  | N/A | 0.31 | 0.11 | 600 | 0 |
| PBC 037 | 83 | F | 127 | 0.7 | N/A | 28 | 20 |  | none | 0.57 | 0.19 | 600 | 0 |
| PBC 038 | 75 | F | 92 | 0.5 | N/A | 21 | 11 |  | N/A | 1.28 | 0.23 | 600 | 0 |
| PBC 039 | 63 | F | 42 | 0.5 | 0.14 | 24 | 15 |  | N/A | 3.57 | 0.14 | 600 | 400 |
| PBC 040 | 77 | F | 72 | 0.7 | N/A | 44 | 30 |  | N/A | 0.50 | 0.16 | 600 | 0 |
| PBC 041 | 57 | M | 66 | 1.2 | N/A | 39 | 30 |  | N/A | 0.59 | 0.12 | 900 | 400 |
| PBC 042 | 58 | F | 120 | 0.8 | N/A | 25 | 18 |  | N/A | 0.63 | 0.14 | 600 | 0 |
| PBC 043 | 62 | F | 78 | 0.6 | N/A | 22 | 16 |  | N/A | 0.55 | 0.14 | 300 | 0 |
| PBC 044 | 51 | F | 154 | 1.1 | N/A | 50 | 66 |  | N/A | 0.63 | 0.24 | 900 | 0 |
| PBC 045 | 54 | F | 256 | 0.6 | 0.62 | 46 | 36 |  | N/A | 0.60 | 0.20 | 900 | 600 |
| PBC 046 | 55 | F | 63 | 0.5 | <0.14 | 41 | 19 |  | N/A | 0.74 | 0.14 | 600 | 400 |
| PBC 047 | 65 | F | 159 | 1.1 | N/A | 22 | 18 |  | N/A | 0.80 | 0.14 | 600 | 0 |
| PBC 048 | 60 | F | 106 | 0.7 | N/A | 17 | 14 |  | N/A | 1.23 | 0.23 | 0 | 0 |
| PBC 049 | 73 | F | 68 | 0.6 | N/A | 21 | 11 |  | N/A | 0.30 | 0.13 | 600 | 400 |
| PBC 050 | 53 | F | 81 | 0.6 | 0.18 | 24 | 22 |  | N/A | 0.57 | 0.18 | 900 | 400 |
| PBC 051 | 64 | F | 43 | 0.4 | N/A | 24 | 11 |  | N/A | 0.56 | 0.18 | 900 | 400 |
| PBC 052 | 86 | F | 130 | 0.6 | N/A | 34 | 15 |  | none | 0.69 | 0.19 | 900 | 0 |
| PBC 053 | 66 | F | 140 | 0.3 | <0.14 | 30 | 19 |  | N/A | 0.58 | 0.15 | 900 | 400 |
| PBC 054 | 49 | F | 90 | 0.7 | N/A | 27 | 14 |  | N/A | 0.92 | 0.10 | 600 | 400 |
| PBC 055 | 80 | M | 131 | 0.7 | N/A | 33 | 38 |  | N/A | 0.30 | 0.10 | 900 | 0 |
| PBC 056 | 68 | F | 145 | 0.7 | N/A | 32 | 27 |  | N/A | 0.78 | 0.17 | 600 | 0 |
| PBC 057 | 71 | F | 110 | 0.8 | N/A | 27 | 23 |  | N/A | 1.11 | 0.25 | 900 | 400 |
| PBC 058 | 53 | F | 42 | 0.6 | 0.645 | 19 | 19 |  | N/A | 0.38 | 0.12 | 600 | 400 |
| PBC 059 | 57 | F | 182 | 0.2 | N/A | 26 | 16 |  | N/A | 0.75 | 0.16 | 600 | 400 |
| PBC 060 | 45 | F | 148 | 1 | 0.022 | 54 | 69 |  | N/A | 0.59 | 0.18 | 600 | 400 |
| PBC 061 | 66 | F | 137 | 0.6 | N/A | 31 | 18 |  | N/A | 0.62 | 0.19 | 900 | 400 |
| PBC 062 | 74 | F | 127 | 0.6 | 0.065 | 18 | 20 |  | N/A | 1.04 | 0.10 | 600 | 0 |
| PBC 063 | 54 | M | 126 | 0.5 | N/A | 36 | 39 |  | N/A | 0.59 | 0.17 | 900 | 400 |
| PBC 064 | 61 | F | 198 | 2.6 | N/A | 49 | 15 |  | N/A | 1.58 | 0.30 | 900 | 400 |
| PBC 065 | 56 | F | 74 | 0.4 | 0.048 | 22 | 15 |  | none | 0.31 | 0.12 | 600 | 400 |
| PBC 066 | 69 | F | 120 | 0.6 | N/A | 32 | 29 |  | N/A | 0.24 | 0.13 | 600 | 0 |
| PBC 067 | 53 | F | 66 | 0.5 | N/A | 30 | 28 |  | N/A | 0.66 | 0.16 | 600 | 0 |
| PBC 068 | 72 | F | 63 | 0.7 | N/A | 21 | 10 |  | N/A | 0.17 | 0.11 | 600 | 200 |
| PBC 069 | 71 | F | 119 | 0.3 | N/A | 16 | 13 |  | N/A | 0.30 | 0.27 | 600 | 0 |
| PBC 070 | 82 | F | 112 | 0.9 | N/A | 23 | 12 |  | N/A | 1.04 | 0.17 | 900 | 0 |
| PBC 071 | 55 | F | 147 | 0.5 | N/A | 34 | 39 |  | N/A | 0.37 | 0.14 | 600 | 200 |
| PBC 072 | 68 | F | 109 | 0.6 | N/A | 25 | 19 |  | N/A | 0.35 | 0.10 | 900 | 0 |
| PBC 073 | 70 | F | 85 | 0.6 | N/A | 28 | 18 |  | N/A | 0.16 | 0.12 | 900 | 0 |
| PBC 074 | 75 | F | 80 | 1.3 | N/A | 36 | 16 |  | N/A | 0.55 | 0.10 | 900 | 400 |
| PBC 075 | 55 | F | 207 | 0.8 | N/A | 27 | 24 |  | N/A | 0.41 | 0.14 | 600 | 0 |
| PBC 076 | 57 | F | 88 | 1.4 | 0.095 | 45 | 26 |  | N/A | 0.16 | 0.14 | 900 | 400 |
| PBC 077 | 71 | F | 104 | 0.5 | N/A | 17 | 10 |  | N/A | 0.50 | 0.17 | 900 | 0 |
| PBC 078 | 70 | M | 105 | 0.7 | N/A | 24 | 24 |  | N/A | 1.34 | 0.21 | 600 | 0 |
| PBC 079 | 71 | M | 47 | 0.4 | N/A | 24 | 24 |  | none | 0.53 | 0.13 | 600 | 400 |
| PBC 080 | 39 | F | 183 | 9.3 | 1.07 | 195 | 100 |  | N/A | 2.63 | 0.43 | 600 | 0 |
| PBC 081 | 60 | F | 140 | 0.6 | 0.15 | 35 | 47 |  | N/A | 0.28 | 0.07 | 0 | 0 |
| PBC 082 | 73 | F | 366 | 0.8 | 0.37 | 60 | 51 |  | N/A | 0.38 | 0.05 | 0 | 0 |
| PBC 083 | 66 | F | 90 | 0.9 | 0.03 | 25 | 21 |  | N/A | 0.51 | 0.06 | 300 | 0 |
| PBC 084 | 57 | F | 183 | 0.8 | 0.03 | 26 | 37 |  | N/A | 0.12 | 0.04 | 0 | 0 |
| PBC 085 | 70 | F | 90 | 0.6 | 0.19 | 65 | 61 |  | N/A | 0.20 | 0.09 | 0 | 0 |
| PBC 086 | 49 | F | 92 | 0.6 | 0.52 | 39 | 53 |  | N/A | 0.99 | 0.05 | 0 | 0 |
| PBC 087 | 48 | F | 723 | 4.8 | 0.14 | 149 | 188 |  | N/A | 1.08 | 0.16 | 0 | 0 |
| PBC 088 | 71 | F | 293 | 0.8 | 0.43 | 69 | 54 |  | N/A | 1.08 | 0.13 | 600 | 0 |
| PBC 089 | 60 | M | 416 | 1.6 | 0.81 | 129 | 127 |  | none | 1.14 | 0.16 | 0 | 0 |
| PBC 090 | 48 | F | 108 | 0.6 | 0.11 | 24 | 20 |  | N/A | 0.33 | 0.08 | 0 | 0 |
| PBC 091 | 52 | F | 276 | 0.5 | 0.63 | 75 | 98 |  | N/A | 0.71 | 0.17 | 0 | 0 |
| PBC 092 | 62 | F | 87 | 0.7 | 0.08 | 23 | 24 |  | N/A | 1.39 | 0.10 | 0 | 0 |
| PBC 093 | 43 | F | 70 | 0.3 | 0.17 | 33 | 61 |  | N/A | 0.43 | 0.07 | 0 | 0 |
| PBC 094 | 44 | F | 141 | 0.7 | 0.05 | 40 | 32 |  | N/A | 0.53 | 0.13 | 0 | 0 |
| PBC 095 | 47 | F | 207 | 0.5 | 0.08 | 39 | 34 |  | N/A | 0.62 | 0.07 | 0 | 0 |
| PBC 096 | 50 | F | 70 | 0.4 | 0.17 | 46 | 76 |  | N/A | 0.78 | 0.10 | 0 | 0 |
| PBC 097 | 63 | F | 326 | 0.8 | 1.15 | 31 | 39 |  | N/A | 0.36 | 0.12 | 600 | 0 |
| PBC 098 | 51 | F | 198 | 0.3 | 0.07 | 29 | 28 |  | N/A | 0.15 | 0.09 | 0 | 0 |
| PBC 099 | 53 | F | 250 | 1 | 0.33 | 77 | 97 |  | none | 0.80 | 0.12 | 600 | 0 |
| PBC 100 | 57 | F | 77 | 1.4 | 0.02 | 22 | 16 |  | N/A | 1.75 | 0.18 | 0 | 0 |
| PBC 101 | 38 | F | 159 | 0.6 | 0.52 | 33 | 49 |  | N/A | 1.08 | 0.12 | 0 | 0 |
| PBC 102 | 68 | F | 271 | 0.4 | 0.32 | 44 | 50 |  | N/A | 1.32 | 0.11 | 0 | 0 |
| PBC 103 | 70 | F | 102 | 0.3 | 1.03 | 28 | 23 |  | N/A | 0.44 | 0.15 | 0 | 0 |
| PBC 104 | 53 | F | 84 | 0.6 | 0.09 | 40 | 38 |  | N/A | 0.95 | 0.38 | 0 | 0 |
| PBC 105 | 69 | F | 143 | 0.7 | 0.05 | 34 | 26 |  | none | 1.34 | 0.14 | 0 | 0 |
| PBC 106 | 37 | F | 89 | 0.7 | 0.14 | 60 | 68 |  | N/A | 1.94 | 0.24 | 0 | 0 |
| PBC 107 | 64 | F | 105 | 0.6 | 0.03 | 53 | 40 |  | N/A | 2.13 | 0.31 | 0 | 0 |
| PBC 108 | 49 | F | 119 | 0.5 | 0.15 | 26 | 25 |  | N/A | 0.68 | 0.19 | 0 | 0 |
| PBC 109 | 73 | M | 151 | 0.8 | 0.64 | 32 | 25 |  | N/A | 1.03 | 0.13 | 0 | 0 |
| PBC 110 | 52 | F | 290 | 0.8 | 0.15 | 43 | 49 |  | N/A | 0.79 | 0.26 | 0 | 0 |
| AIH 01 | 47 | F | 303 | 0.4 | 0.17 | 103 | 111 |  | N/A | 0.60 | 0.12 | 0 | 0 |
| AIH 02 | 74 | F | 160 | 0.6 | 0.36 | 118 | 81 |  | N/A | 1.01 | 0.22 | 0 | 0 |
| AIH 03 | 45 | F | 112 | 0.6 | 0.35 | 122 | 278 |  | N/A | 1.05 | 0.21 | 0 | 0 |
| AIH 04 | 85 | M | 174 | 0.6 | 0.27 | 205 | 292 |  | N/A | 1.08 | 0.15 | 0 | 0 |
| AIH 05 | 56 | F | 117 | 1.1 | 0.28 | 271 | 407 |  | N/A | 0.97 | 0.18 | 0 | 0 |
| AIH 06 | 70 | F | 165 | 1 | 1.06 | 177 | 159 |  | N/A | 3.26 | 0.26 | 0 | 0 |
| AIH 07 | 66 | F | 229 | 1 | 0.3 | 243 | 317 |  | N/A | 0.64 | 0.31 | 300 | 0 |
| AIH 08 | 70 | F | 140 | 0.5 | 0.08 | 105 | 86 |  | N/A | 1.17 | 0.22 | 0 | 0 |
| AIH 09 | 55 | F | 143 | 1 | 0.2 | 686 | 1119 |  | N/A | 1.43 | 0.20 | 0 | 0 |
| AIH 10 | 66 | F | 83 | 0.9 | 0.08 | 131 | 313 |  | N/A | 1.34 | 0.24 | 0 | 0 |
| AIH 11 | 60 | F | 134 | 1.3 | 0.09 | 278 | 383 |  | N/A | 1.33 | 0.15 | 0 | 0 |
| AIH 12 | 25 | F | 170 | 7.7 | 0.13 | 499 | 844 |  | N/A | 0.43 | 0.12 | 0 | 0 |
| AIH 13 | 82 | F | 181 | 0.8 | 0.05 | 198 | 186 |  | N/A | 0.76 | 0.24 | 0 | 0 |
| AIH 14 | 71 | F | 305 | 3.1 | 0.35 | 432 | 688 |  | none | 1.39 | 0.19 | 0 | 0 |
| AIH 15 | 72 | F | 187 | 17.1 | 1.54 | 346 | 599 |  | N/A | 1.40 | 0.25 | 0 | 0 |
| AIH 16 | 50 | F | 118 | 0.6 | 0.04 | 39 | 33 |  | N/A | 1.55 | 0.17 | 0 | 0 |
| AIH 17 | 62 | F | 268 | 8.4 | 0.87 | 992 | 1573 |  | N/A | 1.55 | 0.24 | 0 | 0 |
| AIH 18 | 70 | F | 65 | 0.9 | 0.18 | 902 | 971 |  | none | 1.06 | 0.18 | 0 | 0 |
| AIH 19 | 72 | M | 177 | 1.8 | 0.13 | 1041 | 1550 |  | none | 1.40 | 0.17 | 0 | 0 |
| AIH 20 | 76 | F | 146 | 3.3 | 1.07 | 679 | 493 |  | N/A | 1.14 | 0.19 | 0 | 0 |
| AIH 21 | 72 | M | 226 | 20.1 | 2.13 | 1113 | 987 |  | N/A | 2.89 | 0.40 | 0 | 0 |
| AIH 22 | 49 | F | 102 | 0.8 | 0.03 | 182 | 295 |  | N/A | 0.53 | 0.09 | 0 | 0 |
| AIH 23 | 59 | F | 138 | 0.9 | 0.18 | 265 | 261 |  | N/A | 1.32 | 0.24 | 900 | 0 |
| AIH 24 | 62 | F | 86 | 1.4 | 0.04 | 343 | 489 |  | N/A | 0.57 | 0.18 | 0 | 0 |
| AIH 25 | 81 | F | 73 | 1.1 | 0.06 | 151 | 182 |  | N/A | 0.55 | 0.21 | 0 | 0 |
| IgG4-SC 01 | M | 48 | N/A | N/A | N/A | N/A | N/A |  | N/A | 0.88 | 0.15 | N/A | N/A |
| IgG4-SC 02 | M | 54 | N/A | N/A | N/A | N/A | N/A |  | N/A | 2.42 | 0.29 | N/A | N/A |
| IgG4-SC 03 | M | 52 | N/A | N/A | N/A | N/A | N/A |  | N/A | 1.05 | 0.10 | N/A | N/A |
| IgG4-SC 04 | M | 75 | N/A | N/A | N/A | N/A | N/A |  | N/A | 0.65 | 0.45 | N/A | N/A |
| IgG4-SC 05 | M | 60 | N/A | N/A | N/A | N/A | N/A |  | N/A | 0.73 | 0.10 | N/A | N/A |
| IgG4-SC 06 | M | 63 | N/A | N/A | N/A | N/A | N/A |  | N/A | 0.41 | 0.10 | N/A | N/A |
| IgG4-SC 07 | M | 68 | N/A | N/A | N/A | N/A | N/A |  | N/A | 0.42 | 0.14 | N/A | N/A |
| IgG4-SC 08 | M | 67 | N/A | N/A | N/A | N/A | N/A |  | N/A | 0.28 | 0.10 | N/A | N/A |
| IgG4-SC 09 | M | 66 | N/A | N/A | N/A | N/A | N/A |  | N/A | 0.66 | 0.10 | N/A | N/A |
| IgG4-SC 10 | M | 71 | N/A | N/A | N/A | N/A | N/A |  | N/A | 0.80 | 0.32 | N/A | N/A |
| IgG4-SC 11 | M | 64 | N/A | N/A | N/A | N/A | N/A |  | N/A | 1.32 | 0.40 | N/A | N/A |
| IgG4-SC 12 | M | 64 | N/A | N/A | N/A | N/A | N/A |  | N/A | 0.61 | 0.09 | N/A | N/A |
| IgG4-SC 13 | M | 70 | N/A | N/A | N/A | N/A | N/A |  | N/A | 0.96 | 0.17 | N/A | N/A |
| IgG4-SC 14 | M | 74 | N/A | N/A | N/A | N/A | N/A |  | N/A | 0.41 | 0.13 | N/A | N/A |
| IgG4-SC 15 | M | 61 | N/A | N/A | N/A | N/A | N/A |  | N/A | 0.35 | 0.21 | N/A | N/A |
| IgG4-SC 16 | F | 55 | N/A | N/A | N/A | N/A | N/A |  | N/A | 0.73 | 0.14 | N/A | N/A |
| IgG4-SC 17 | M | 49 | N/A | N/A | N/A | N/A | N/A |  | N/A | 0.42 | 0.14 | N/A | N/A |
| IgG4-SC 18 | M | 80 | N/A | N/A | N/A | N/A | N/A |  | N/A | 0.82 | 0.12 | N/A | N/A |
| IgG4-SC 19 | M | 62 | N/A | N/A | N/A | N/A | N/A |  | N/A | 0.68 | 0.12 | N/A | N/A |
| IgG4-SC 20 | M | 72 | N/A | N/A | N/A | N/A | N/A |  | N/A | 0.79 | 0.15 | N/A | N/A |
| IgG4-SC 21 | M | 65 | N/A | N/A | N/A | N/A | N/A |  | N/A | 0.84 | 0.11 | N/A | N/A |
| IgG4-SC 22 | M | 65 | N/A | N/A | N/A | N/A | N/A |  | N/A | 0.90 | 0.13 | N/A | N/A |
| IgG4-SC 23 | M | 71 | N/A | N/A | N/A | N/A | N/A |  | N/A | 0.28 | 0.05 | N/A | N/A |
| IgG4-SC 24 | F | 68 | N/A | N/A | N/A | N/A | N/A |  | N/A | 0.63 | 0.13 | N/A | N/A |
| IgG4-SC 25 | M | 66 | N/A | N/A | N/A | N/A | N/A |  | N/A | 0.32 | 0.12 | N/A | N/A |
| IgG4-SC 26 | M | 73 | N/A | N/A | N/A | N/A | N/A |  | N/A | 0.23 | 0.06 | N/A | N/A |
| IgG4-SC 27 | M | 77 | N/A | N/A | N/A | N/A | N/A |  | N/A | 0.40 | 0.09 | N/A | N/A |
| IgG4-SC 28 | M | 39 | N/A | N/A | N/A | N/A | N/A |  | N/A | 0.43 | 0.06 | N/A | N/A |
| IgG4-SC 29 | M | 49 | N/A | N/A | N/A | N/A | N/A |  | N/A | 0.92 | 0.15 | N/A | N/A |
| IgG4-SC 30 | M | 75 | N/A | N/A | N/A | N/A | N/A |  | N/A | 0.63 | 0.11 | N/A | N/A |
| IgG4-SC 31 | M | 74 | N/A | N/A | N/A | N/A | N/A |  | N/A | 0.45 | 0.07 | N/A | N/A |
| IgG4-SC 32 | M | 70 | N/A | N/A | N/A | N/A | N/A |  | N/A | 2.17 | 0.12 | N/A | N/A |
| IgG4-SC 33 | M | 62 | N/A | N/A | N/A | N/A | N/A |  | N/A | 1.30 | 0.10 | N/A | N/A |
| IgG4-SC 34 | M | 78 | N/A | N/A | N/A | N/A | N/A |  | N/A | 0.35 | 0.05 | N/A | N/A |
| IgG4-SC 35 | M | 67 | N/A | N/A | N/A | N/A | N/A |  | N/A | 1.06 | 0.13 | N/A | N/A |
| IgG4-SC 36 | F | 59 | N/A | N/A | N/A | N/A | N/A |  | N/A | 0.56 | 0.09 | N/A | N/A |
| IgG4-SC 37 | F | 70 | N/A | N/A | N/A | N/A | N/A |  | N/A | 1.11 | 0.08 | N/A | N/A |
| IgG4-SC 38 | M | 76 | N/A | N/A | N/A | N/A | N/A |  | N/A | 0.46 | 0.11 | N/A | N/A |
| IgG4-SC 39 | F | 66 | N/A | N/A | N/A | N/A | N/A |  | N/A | 0.50 | 0.15 | N/A | N/A |
| IgG4-SC 40 | M | 59 | N/A | N/A | N/A | N/A | N/A |  | N/A | 1.20 | 0.15 | N/A | N/A |
| IgG4-SC 41 | M | 78 | N/A | N/A | N/A | N/A | N/A |  | N/A | 0.71 | 0.07 | N/A | N/A |
| IgG4-SC 42 | M | 70 | N/A | N/A | N/A | N/A | N/A |  | N/A | 1.11 | 0.14 | N/A | N/A |
| IgG4-SC 43 | M | 76 | N/A | N/A | N/A | N/A | N/A |  | N/A | 0.68 | 0.09 | N/A | N/A |
| IgG4-SC 44 | F | 69 | N/A | N/A | N/A | N/A | N/A |  | N/A | 0.48 | 0.14 | N/A | N/A |
| IgG4-SC 45 | M | 72 | N/A | N/A | N/A | N/A | N/A |  | N/A | 0.38 | 0.13 | N/A | N/A |
| IgG4-SC 46 | M | 62 | N/A | N/A | N/A | N/A | N/A |  | N/A | 0.30 | 0.08 | N/A | N/A |
| IgG4-SC 47 | M | 63 | N/A | N/A | N/A | N/A | N/A |  | N/A | 0.57 | 0.12 | N/A | N/A |
| IgG4-SC 48 | F | 47 | N/A | N/A | N/A | N/A | N/A |  | N/A | 0.59 | 0.08 | N/A | N/A |
| IgG4-SC 49 | M | 69 | N/A | N/A | N/A | N/A | N/A |  | N/A | 0.59 | 0.11 | N/A | N/A |
| IgG4-SC 50 | F | 65 | N/A | N/A | N/A | N/A | N/A |  | N/A | 0.59 | 0.13 | N/A | N/A |
| IgG4-SC 51 | M | 77 | N/A | N/A | N/A | N/A | N/A |  | N/A | 0.55 | 0.10 | N/A | N/A |
| IgG4-SC 52 | M | 72 | N/A | N/A | N/A | N/A | N/A |  | N/A | 0.38 | 0.11 | N/A | N/A |
| IgG4-SC 53 | M | 75 | N/A | N/A | N/A | N/A | N/A |  | N/A | 7.25 | 0.42 | N/A | N/A |
| IgG4-SC 54 | M | 61 | N/A | N/A | N/A | N/A | N/A |  | N/A | 0.25 | 0.10 | N/A | N/A |
| IgG4-SC 55 | M | 66 | N/A | N/A | N/A | N/A | N/A |  | N/A | 1.27 | 0.11 | N/A | N/A |
| IgG4-SC 56 | F | 90 | N/A | N/A | N/A | N/A | N/A |  | N/A | 0.71 | 0.12 | N/A | N/A |
| IgG4-SC 57 | F | 65 | N/A | N/A | N/A | N/A | N/A |  | N/A | 0.56 | 0.15 | N/A | N/A |
| IgG4-SC 58 | F | 62 | N/A | N/A | N/A | N/A | N/A |  | N/A | 0.36 | 0.11 | N/A | N/A |
| IgG4-SC 59 | F | 73 | N/A | N/A | N/A | N/A | N/A |  | N/A | 0.43 | 0.07 | N/A | N/A |
| IgG4-SC 60 | M | 72 | N/A | N/A | N/A | N/A | N/A |  | N/A | 1.55 | 0.14 | N/A | N/A |
| IgG4-SC 61 | F | 45 | N/A | N/A | N/A | N/A | N/A |  | N/A | 0.34 | 0.08 | N/A | N/A |
| IgG4-SC 62 | M | 71 | N/A | N/A | N/A | N/A | N/A |  | N/A | 1.00 | 0.11 | N/A | N/A |
| IgG4-SC 63 | M | 49 | N/A | N/A | N/A | N/A | N/A |  | N/A | 0.86 | 0.13 | N/A | N/A |
| IgG4-SC 64 | M | 65 | N/A | N/A | N/A | N/A | N/A |  | N/A | 0.70 | 0.12 | N/A | N/A |
| IgG4-SC 65 | M | 44 | N/A | N/A | N/A | N/A | N/A |  | N/A | 0.33 | 0.10 | N/A | N/A |
| IgG4-SC 66 | M | 77 | N/A | N/A | N/A | N/A | N/A |  | N/A | 0.53 | 0.12 | N/A | N/A |
| IgG4-SC 67 | M | 72 | N/A | N/A | N/A | N/A | N/A |  | N/A | 0.47 | 0.13 | N/A | N/A |
| IgG4-SC 68 | M | 79 | N/A | N/A | N/A | N/A | N/A |  | N/A | 0.62 | 0.14 | N/A | N/A |
| IgG4-SC 69 | M | 54 | N/A | N/A | N/A | N/A | N/A |  | N/A | 1.31 | 0.20 | N/A | N/A |
| IgG4-SC 70 | F | 74 | N/A | N/A | N/A | N/A | N/A |  | N/A | 0.58 | 0.13 | N/A | N/A |
| IgG4-SC 71 | F | 83 | N/A | N/A | N/A | N/A | N/A |  | N/A | 1.21 | 0.14 | N/A | N/A |
| IgG4-SC 72 | M | 42 | N/A | N/A | N/A | N/A | N/A |  | N/A | 1.99 | 0.18 | N/A | N/A |
| IgG4-SC 73 | M | 80 | N/A | N/A | N/A | N/A | N/A |  | N/A | 0.45 | 0.08 | N/A | N/A |
| IgG4-SC 74 | M | 63 | N/A | N/A | N/A | N/A | N/A |  | N/A | 0.52 | 0.13 | N/A | N/A |
| IgG4-SC 75 | M | 79 | N/A | N/A | N/A | N/A | N/A |  | N/A | 0.65 | 0.12 | N/A | N/A |
| IgG4-SC 76 | F | 62 | N/A | N/A | N/A | N/A | N/A |  | N/A | 0.43 | 0.17 | N/A | N/A |
| IgG4-SC 77 | M | 76 | N/A | N/A | N/A | N/A | N/A |  | N/A | 0.59 | 0.15 | N/A | N/A |
| IgG4-SC 78 | M | 65 | N/A | N/A | N/A | N/A | N/A |  | N/A | 0.26 | 0.04 | N/A | N/A |
| IgG4-SC 79 | F | 61 | N/A | N/A | N/A | N/A | N/A |  | N/A | 0.54 | 0.09 | N/A | N/A |
| IgG4-SC 80 | M | 76 | N/A | N/A | N/A | N/A | N/A |  | N/A | 0.61 | 0.13 | N/A | N/A |
| IgG4-SC 81 | F | 75 | N/A | N/A | N/A | N/A | N/A |  | N/A | 0.46 | 0.07 | N/A | N/A |
| IgG4-SC 82 | M | 75 | N/A | N/A | N/A | N/A | N/A |  | N/A | 1.56 | 0.18 | N/A | N/A |
| IgG4-SC 83 | F | 73 | N/A | N/A | N/A | N/A | N/A |  | N/A | 0.44 | 0.11 | N/A | N/A |
| IgG4-SC 84 | M | 63 | N/A | N/A | N/A | N/A | N/A |  | N/A | 1.02 | 0.06 | N/A | N/A |
| IgG4-SC 85 | M | 55 | N/A | N/A | N/A | N/A | N/A |  | N/A | 1.00 | 0.14 | N/A | N/A |
| IgG4-SC 86 | F | 59 | N/A | N/A | N/A | N/A | N/A |  | N/A | 1.08 | 0.15 | N/A | N/A |
| IgG4-SC 87 | M | 68 | N/A | N/A | N/A | N/A | N/A |  | N/A | 0.60 | 0.10 | N/A | N/A |
| IgG4-SC 88 | M | 77 | N/A | N/A | N/A | N/A | N/A |  | N/A | 0.63 | 0.16 | N/A | N/A |
| IgG4-SC 89 | M | 56 | N/A | N/A | N/A | N/A | N/A |  | N/A | 2.81 | 0.10 | N/A | N/A |
| IgG4-SC 90 | M | 71 | N/A | N/A | N/A | N/A | N/A |  | N/A | 0.44 | 0.08 | N/A | N/A |
| IgG4-SC 91 | M | 64 | N/A | N/A | N/A | N/A | N/A |  | N/A | 0.27 | 0.11 | N/A | N/A |
| IgG4-SC 92 | M | 70 | N/A | N/A | N/A | N/A | N/A |  | N/A | 0.24 | 0.08 | N/A | N/A |
| IgG4-SC 93 | M | 67 | N/A | N/A | N/A | N/A | N/A |  | N/A | 0.35 | 0.07 | N/A | N/A |
| IgG4-SC 94 | M | 72 | N/A | N/A | N/A | N/A | N/A |  | N/A | 0.38 | 0.08 | N/A | N/A |
| IgG4-SC 95 | M | 79 | N/A | N/A | N/A | N/A | N/A |  | N/A | 0.57 | 0.08 | N/A | N/A |
| SSC recurrent pyogenic cholangitis 1 | F | 78 | 84.35 | 0.7 | 0.6 | 21 | 12 |  | N/A | 1.29 | 0.12 | 600 | 0 |
| SSC recurrent pyogenic cholangitis 2 | M | 44 | 140 | 1.9 | 13.57 | 21 | 92 |  | N/A | 0.53 | 0.07 | 0 | 0 |
| SSC recurrent pyogenic cholangitis 3 | F | 89 | 151 | 0.5 | 0.47 | 18 | 13 |  | N/A | 0.57 | 0.07 | 0 | 0 |
| SSC anastomotic stenosis 1 | M | 49 | 577.9 | 1 | 2.1 | 74 | 76 |  | none | 1.01 | 0.05 | 600 | 0 |
| SSC anastomotic stenosis 2 | M | 62 | 175 | 0.4 | 0 | 23 | 20 |  | N/A | 0.65 | 0.07 | 200 | 0 |
| SSC Mirizzi syndrome | M | 74 | 142.5 | 0.7 | 0.2 | 25 | 69 |  | N/A | 0.42 | 0.05 | 0 | 0 |
| Eosinophilic cholangitis | F | 80 | 750 | 1 | 2.18 | 86 | 147 |  | none | 0.39 | 0.04 | 0 | 0 |
| irAE  cholangitis 1 | M | 63 | 257 | 1.2 | 2.03 | 32 | 85 |  | N/A | 0.34 | 0.04 | 900 | 0 |
| irAE  cholangitis 2 | F | 63 | 1733 | 1.3 | 0.88 | 381 | 306 |  | N/A | 0.51 | 0.04 | 0 | 0 |
| irAE  cholangitis 3 | M | 72 | 407 | 1.1 | 1.57 | 50 | 37 |  | N/A | 0.27 | 0.05 | 900 | 0 |
| irAE  cholangitis 4 | F | 75 | 1625 | 0.5 | 2.57 | 74 | 90 |  | N/A | 0.33 | 0.04 | 0 | 0 |
| ※ IBD was confirmed through colonoscopy, and "N/A" indicates that a colonoscopy was not performed or that it is not known whether a colonoscopy was performed. | | | | | | | | | | | | | |
| Abbreviations: ALP, alkaline phosphatase; T-bil, total bilirubin; CRP, C-reactive protein; AST, aspartate aminotransferase; ALT, Alanine aminotransferase; N/A, not available; M, male; F, female; ELISA, enzyme-linked immunosorbent assay; PSC, primary sclerosing cholangitis; CCA, cholangiocarcinoma; PBC, primary biliary cholangitis; AIH, autoimmune hepatitis; IgG4-SC, Immunoglobulin G4-related sclerosing cholangitis; SSC, secondary sclerosing cholangitis; irAE, immune-related adverse event; IBD, inflammatory bowel disease; UC, ulcerative colitis; CD, Crohn’s disease; IBD-U, unclassified-inflammatory bowel disease | | | | | | | | | | | | | |

Supplemental Table 2. Comparison of clinical characteristics between anti-integrin αvβ6 autoantibody-positive and -negative in PSC patients without IBD

|  | Anti-integrin αvβ6 autoantibody | |  |
| --- | --- | --- | --- |
|  | Positive n=7 | Negative n=5 | P-value |
| Number of PSC-specific features | 3 | 3 | 0.951 |
| T-bil | 0.6 | 0.7 | 0.389 |
| ALP | 145 | 204 | 0.730 |
| CRP | 0.3 | 0.075 | 0.486 |
| ALT | 31 | 39 | 0.778 |
| Values are medians. |  |  |  |
| PSC, primary sclerosing cholangitis; IBD, inflammatory bowel disease; T-bil, total bilirubin; ALP, alkaline phosphatase; CRP, C-reactive protein; ALT, alanine aminotransferase; UC, ulcerative colitis | | | |
